# Promoting salt tolerance, growth, and phytochemical responses in coriander (*Coriandrum sativum* L. cv. Balady) via eco-friendly *Bacillus subtilis* and cobalt

**DOI:** 10.1186/s12870-024-05517-3

**Published:** 2024-09-10

**Authors:** Sary H. Brengi, Maneea Moubarak, Hany M. El-Naggar, Amira R. Osman

**Affiliations:** 1 Department of Horticulture, Faculty of Agriculture, Damanhour University, Damanhour, Beheira 22516 Egypt; 2 Department of Floriculture, Faculty of Agriculture, Alexandria University (El-Shatby), Alexandria, 21545 Egypt

**Keywords:** Endophytic microorganism, Micronutrient of cobalamin, Medicinal and aromatic plant, Oxidative stress, Salinity

## Abstract

In plant production, evaluation of salt stress protectants concerning their potential to improve growth and productivity under saline stress is critical. *Bacillus subtilis* (Bs) and cobalt (Co) have been proposed to optimize salt stress tolerance in coriander (*Coriandrum sativum* L. cv. Balady) plants by influencing some physiological activities. The main aim of this work is to investigate the response of (Bs) and (Co) as eco-safe salt stress protectants to resist the effect of salinity, on growth, seed, and essential oil yield, and the most important biochemical constituents of coriander produced under salt stress condition. Therefore, in a split-plot factorial experiment design in the RCBD (randomized complete block design), four levels of salinity of NaCl irrigation water (SA) were assigned to the main plots; (0.5, 1.5, 4, and 6 dS m^−1^); and six salt stress protectants (SP) were randomly assigned to the subplots: distilled water; 15 ppm (Co1); 30 ppm (Co2); (Bs); (Co1 + Bs); (Co2 + Bs). The study concluded that increasing SA significantly reduced coriander growth and yield by 42.6%, which could be attributed to ion toxicity, oxidative stress, or decreased vital element content. From the results, we recommend that applying Bs with Co (30 ppm) was critical for significantly improving overall growth parameters. This was determined by the significant reduction in the activity of reactive oxygen species scavenging enzymes: superoxide dismutase (SOD), catalase (CAT), and malondialdehyde (MDA) and non-enzyme: proline by 5, 11.3, 14.7, and 13.8% respectively, while increasing ascorbic acid by 8% and preserving vital nutrient levels and enhancing plant osmotic potential to buffer salt stress, seed yield per plant, and essential oil yield increased by 12.6 and 18.8% respectively. The quality of essential oil was indicated by highly significant quantities of vital biological phytochemicals such as linalool, camphor, and protein which increased by 10.3, 3.6, and 9.39% respectively. Additional research is suggested to determine the precise mechanism of action of Bs and Co's dual impact on medicinal and aromatic plant salt stress tolerance.

**Supplementary Information:**

The online version contains supplementary material available at 10.1186/s12870-024-05517-3.

## Introduction

Climate change has recently been linked to a significant increase in salt stress. Thus saline irrigation water has a severe negative impact on agricultural, horticultural, medicinal, and aromatic plant productivity globally and reduced the potential yield of annual crops by around fifty percent [1, 2]. Salt stress promotes abiotic stress, which results in water deficiency; a lack of essential nutrients such as K^+^; and Na^+^ toxicity inside plants, which causes decreased photosynthetic efficiency and biomass accumulation; PAL gene expression; stomatal closure; reduced leaf expansion; and antioxidant defense systems, all of which influence important molecules involved in medicinal plant synthesis, such as the total phenolic content, lipids, proteins, and DNA; thus, salt stress affects plant production quality [3, 4]. As a result, new approaches for dealing with the threat of global salt stress to agricultural production are needed. Several solutions, including the genetic selection of plant cultivars that are most tolerant species to salt stress, plant genetic engineering, and the introduction of plant growth-promoting bacteria (PGPB), have been devised to mitigate the detrimental effects of high salt concentrations on plant growth [5, 6].

*Bacillus subtilis* bacteria are among the best-performing endophytic microorganism eco-safe organisms and may influence plant growth and create systemic resistance to abiotic stresses, such as drought, salinity, and heavy metals, through their participation in antioxidant activities and phenylpropanoid metabolism, optimizing the synthesis of plant hormones such as auxins and gibberellins, or serving as plant protection agents against microbial infections [7]. The stimulating effect of these plants by endophytic microorganisms may influence the plant to be a limitless source of novel metabolites, the application of which could reduce agrochemicals, thus improving agricultural, horticultural, medicinal, and aromatic plants; furthermore, such plants could be used as base materials in a multitude of categories, such as cosmetics, pharmaceuticals, and food industry production [8, 9]. *B. subtilis* has been proven to protect numerous medicinal and aromatic plant species from various abiotic stressors, including sweet basil [10, 11], peppermint [12], Jew’s Mallow [13], turmeric [14], rosemary [15], and ginger [16].

Cobalt is an essential micronutrient of cobalamin (vitamin B_12_) that is a cofactor for a wide variety of enzymes and an essential component of several proteins in prokaryotes and mammals. Reduced activity of this enzyme can cause megaloblastic anemia [17]. Co can alleviate abiotic stressors such as salt and drought by acting as a component of several enzymes and coenzymes, positively affecting plant development and metabolism, regulating ion homeostasis, and modifying phytohormones at low concentrations [18–22]. However, the impact of Co on redox equilibrium under salt stress has received little attention. Cobalt and salt together enhance the stress tolerance of several medicinal and aromatic plant species, including *Salvia officinalis* L. [23], barley and wheat [24], and *Pennisetum divisum* [25].

Coriander (*Coriandrum sativum* L.) is an annual herb in the Apiaceae (Umbelliferae) family. It is a very essential medicinal and fragrant herbaceous plant that has been used extensively in folk medicine for centuries as a digestive system regulator, diuretic, antibacterial, vegetable, appetizer, and condiment [26, 27]. The plant's green or dry leaves are used in daily human food as a condiment for flavoring salads, soups, various pastries, pickles and seeds (fruit) are used to flavor falafel, which is a traditional Egyptian dish [9]. Coriander is a medicinal plant that contains a high concentration of essential oils in its roots, leaves, stems, flowers, fruits, and seeds. One of the greatest intriguing options in the culinary, agrochemical, pesticide, cosmetics, and pharmaceutical industries is coriander essential oil, which contains important biological components such as borneol, camphor, cineole, geranyl acetate, coriandrol, cymene, dipentene, pinene geraniol, linalool, phellandrene terpineol, terpinene, and terpinolene [28–30]. Furthermore, they significantly affect plant tolerance to various stress environments [31]. It is also famous for its high concentration of medicinal agents, such as anticonvulsants, antidiabetic, antimicrobial, antioxidant, and other compounds, which protect human body cells from sickness [32, 33].

Therefore, the current research was designed to investigate the response of *Bacillus subtilis* (Bs) and cobalt (Co) [CoSO_4_.7 H_2_O] as eco-safe salt stress protectants to resist the effect of salinity, on growth, seed and essential oil yield, and the most important biochemical constituents of coriander produced under salt stress condition.

## Materials and methods

### Cultivation and plant material

**Fig. 1:**
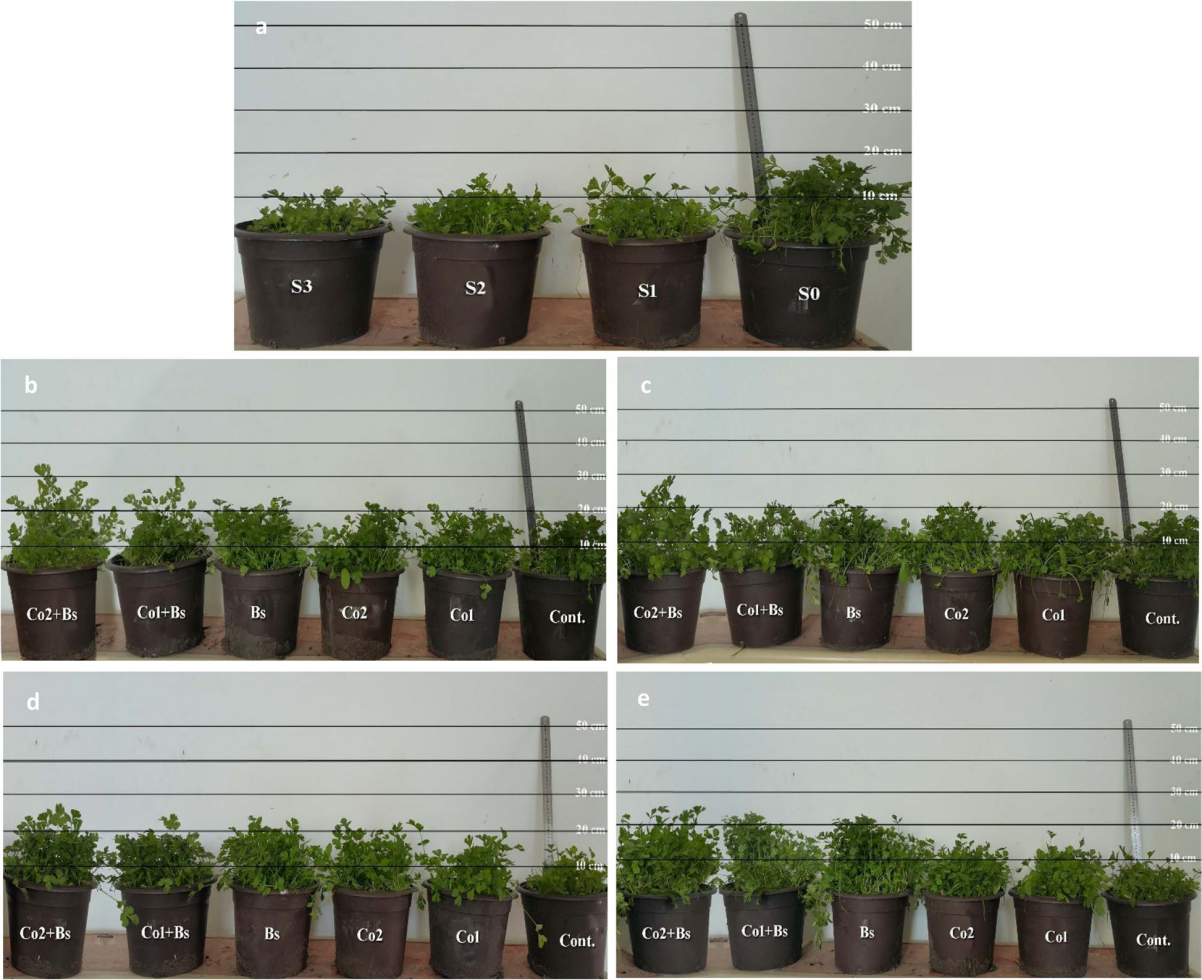
**a** The main effect of the four levels of NaCl irrigation water salinity (SA): tap water as a control S0, 0.5; S1, 1.5; S2, 4; and S3, 6 dS m.^−1^. **b**,** c**,** d**, and** e** The interaction between the four salinity levels (S0, S1, S2, and S3) respectively and the six salt stress protectants: distilled water Cont.; 15 ppm (Co1); 30 ppm (Co2); (Bs); (Co1 + Bs); (Co2 + Bs) 50 days after seed sowing of *Coriandrum sativum* L. cv. Balady. Where: the control (Cont.), *B. subtilis* (Bs) and cobalt (Co)

To determine the efficacy of using various eco-safe salt stress protectants to reduce the deleterious effects of salinity stress on growth parameters, characteristics, chlorophyll content, dry leaf content, K:Na ratio, electrolyte leakage, antioxidant enzyme activity, Asco, proline content in leaves, some biological phytochemicals in seed oil, and seed and oil yield parameters of coriander plants, two pot experiments were conducted on a private farm in Abu Hommus, El-Beheira Governorate, Egypt (31° 5′ 35" north, 30° 18′ 51" east), during two successive winter seasons, from October to April 2022 and 2023. The local variety of coriander seeds (*Coriandrum sativum* L., cv. Balady) was obtained from the Agriculture Administration Abu Hommus, El-Beheira Governorate, Egypt. The seeds were uniformly sized, and five seeds were seeded in black plastic pots (30 cm in diameter × 35 cm in height) filled with 10 kg of soil Fig. 1. The best three plants were preserved after germination, and the physio-chemical properties of the soil were analyzed before planting [34, 35] (Table 1).

**Table 1:** Physical and chemical characteristics of the experimental soil during the two seasons

**Season 1**								
**Sand**	**Silt**	**Clay**	**Texture**	**Bulk density** **(g cm**^**−3**^**)**	**pH**	**EC** **(dSm**^**−1**^**)**	**Organic matter (%)**	**CaCO**_**3**_** (%)**
22	21.6	56.4	Clay	1.54	8.1	0.74	1.45	1.75
			**DTPA-extractable (ppm)**				**Soluble Ions (meq/L)**	
**N %**	**P (ppm)**	**Exchangeable K (ppm)**	**Zn**	**Mn**	**Fe**	**Cu**	**Na**	**Cl**
0.1	39	205	2.1	43	28	3.2	4	3.2
**Season 2**								
**Sand**	**Silt**	**Clay**	**Texture**	**Bulk density** **(g cm**^**−3**^**)**	**pH**	**EC** **(dSm**^**−1**^**)**	**Organic matter (%)**	**CaCO**_**3**_ **(%)**
23.5	23.7	52.8	Clay	1.52	8.2	0.86	1.34	1.83
			**DTPA-extractable (ppm)**				**Soluble Ions (meq/L)**	
**N %**	**P (ppm)**	**Exchangeable K (ppm)**	**Zn**	**Mn**	**Fe**	**Cu**	**Na**	**Cl**
0.092	36	198	1.97	37	24	3.6	5.6	6.9

### Experimental design and treatments

Two factors in a split-plot factorial experiment were arranged by a randomized complete block design with five replications. The main plots were four salinity levels (SA); tap water as a control (Cont.) 0.5, 1.5, 4, and 6 dS m^−1^, and subplots were the six eco-safe salt stress protectants (SP): distilled water as a control (Cont.), cobalt [CoSO_4_.7 H_2_O] (Merck Life Science Ltd. Cairo, Egypt) (Co1) at 15 ppm, (Co2) at 30 ppm, and *Bacillus subtilis* (Bs) (Microbial Culture Network Ain Shams University Cairo, Egypt) at 0.4 L per hectare (8 × 10^8^ spores mL^−1^), (Co1 + Bs), and (Co2 + Bs). All possible combinations of two factors were performed (4 × 6 = 24 treatments and 120 experimental units) (Table S1). The treatments were applied with irrigation one week after sowing the coriander seeds and one week after each harvest, using the same amount of water. The Ministry of Agriculture's instructions for cultivating coriander plants in clay loam soil included supplemental fertilizer requirements and agricultural strategies necessary for its growth were applied.

### Growth parameters, characteristics, and photosynthetic pigment concentrations in fresh leaves

After 50 days after sowing, the following data were recorded: the number of leaves (NL), leaf length (LL) (cm), shoot fresh weight (SFW) (g), root fresh weight (RFW) (g), root dry weight (RDW) (g), plant fresh weight (PFW) (g), plant dry weight (PDW) (g), plant height (Ph) (cm), and chlorophyll content (SPAD Unit) using a chlorophyll meter (SPAD-502 m, Konica Minolta, Japan). Dry weights were measured after drying the vegetative parts and roots for 72 h at a temperature of 70°C.

### Element content, K:Na ratio, electrolyte leakage, and protein content

Nine plants were picked, cleansed with tap water, and subsequently rinsed three times with distilled water. The samples were subsequently dried in a forced-air oven at 70°C until they reached a consistent weight. Finally, the samples were ground in a Willy mill and filtered using a 30-mesh screen. A total of 0.2 g of dried fine powder from the sampled leaves was digested with a combination of hydrogen peroxide and sulfuric acid (El Gomhoria for Medicines and Medical Supplies Trading Alexandria, Egypt.) [36]. Kjeldahl's approach was used to calculate the percentage of total nitrogen (N) in dry leaves utilizing mineral analysis [37]. The amounts of potassium (K), phosphorus (P), sodium (Na), and chloride (Cl) were determined using the methods described by Cottenie et al. [38]. The protein concentration was measured according to Krul [39]. Electrolyte leakage was utilized to determine the integrity of the cell membranes, and the measurements were performed following the approach of Lutts et al. [40].

### Determination of the activity of antioxidant enzymes, total protein content, Asco, and proline content in leaves

Gao et al. [41] studied the activity of the enzyme superoxide dismutase (SOD) (EC 1.15.1.1). The ideal amount of enzyme extract required to produce 50% inhibition of photochemical degradation of NBT was found to be one unit of SOD activity. According to Aebi [42], the elimination of H_2_O_2_ from crude extract samples revealed catalase (CAT) activity (EC 1.11.1.6). The activity of lipid peroxidation was determined by measuring the levels of malondialdehyde (MDA) using a technique proposed by Gérard-Monnier et al. [43]. The entire set of enzymes was obtained using the method provided by Agarwal et al. [44]. In particular, 100 mg of freshly collected coriander leaves was crushed in an extraction buffer cooled with ice. Bovine serum albumin (Sigma-Aldrich, Inc. St. Louis, MO. United States) was utilized as a standard for determining the total protein content of the raw extract [45]. A spectrophotometer (Unico W49376 Spectrophotometer 1200, Shanghai, China) with a 525 nm wavelength was used to detect the presence of ascorbic acid in the supernatants. A standard curve was created using ascorbic acid as an analytical reagent from Solarbio. The quantity of ascorbic acid is represented as milligrams per 100 g of fresh weight using the approach given by Srivastava and Singh [46]. The proline content of the leaves was measured using the approach proposed by Bates et al. [47].

### Determination of biological phytochemicals in seed oil, seed yield per plant, leaf oil, and essential oil yield

After the seeds matured, a representative sample of six random plants was randomly selected from each experimental unit to calculate the seed yield per plant (SYP). The percentage of essential oil yield (EOYS) consumed was measured using a Clevenger apparatus according to Guenther [48], and the following oil phytochemicals were measured: linalool (%), γ-terpinene (%), α-pinene (%), p-cymene (%), camphor (%), and geranyl acetate (%) using Agilent Gas Chromatography-Mass Spectrometry (GC‒MS) according to El-Kinany et al. [49]. The phytochemicals of the essential oils were determined by their retention time and correlation of mass spectra with those of the NIST and Wiley library databases. Leaf essential oil (LO) was extracted according to the approach of El-Massry et al. [50]. Fresh coriander leaves were cut into small fragments and subjected to water distillation for three hours using Clevenger equipment. The essential oils were collected, dehydrated with anhydrous sodium sulfate, and stored in sealed glass vials lined with aluminum foil at 20°C.

### Statistical analysis and experimental design

In our experiment, the design was established as a split-plot design in the RCBD (randomized complete block design), where NaCl levels were assigned to the main plots, and the protective treatments were randomly assigned to the subplots. The experiment included 24 treatments that consisted of four NaCl concentrations and six protective treatments, five replicates (five pots for each treatment, with three plans for each pot). All the data were analyzed statistically by the CoStat statistical software program version 6.4; Co Hort, USA [51] and are presented as the average of the two seasons. At a probability level of *p* < 0.05, the means were compared using the least significant difference (LSD) analysis.

## Results

### Plant growth parameters, morphology, and chlorophyll content

**Fig. 2:**
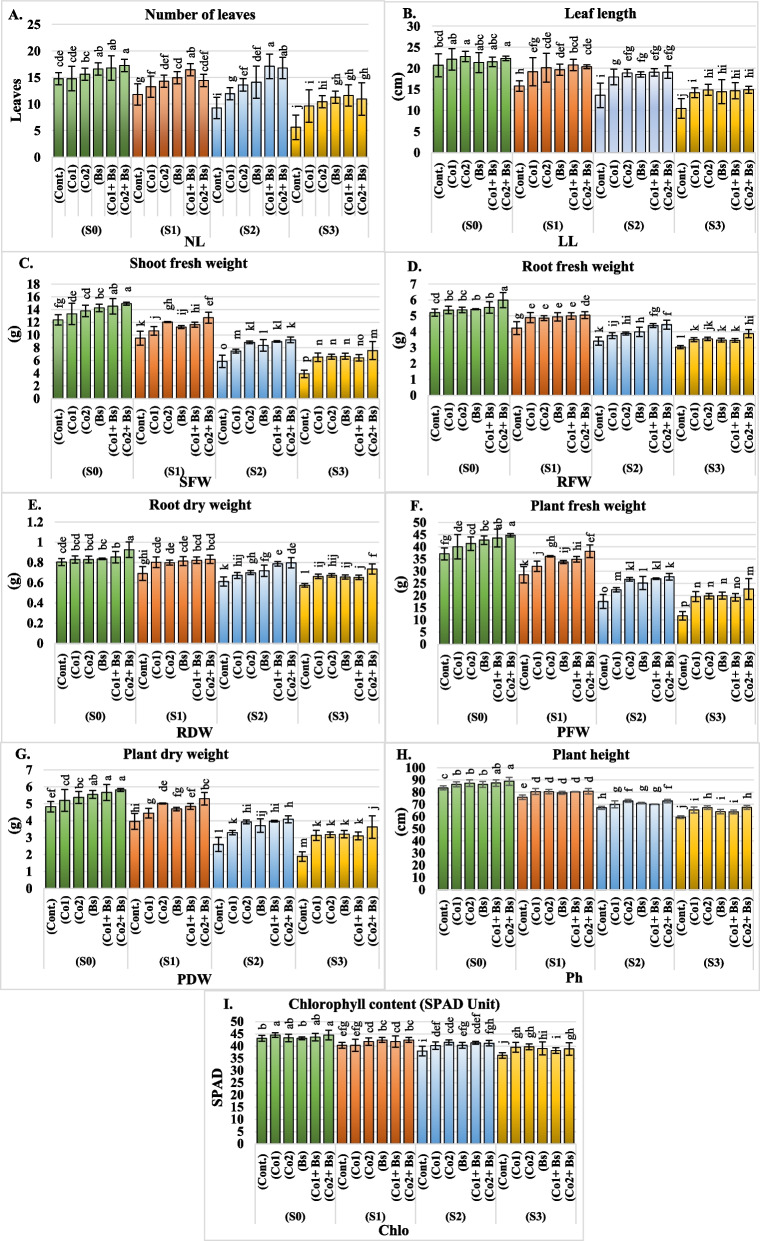
Growth parameters and characteristics of *Coriandrum sativum* L. cv. Balady; **A** number of leaves “NL”, **B** leaf length “LL” (cm), **C** shoot fresh weight “SFW” (g), **D** root fresh weight “RFW” (g), **E** root dry weight “RDW” (g), **F** plant fresh weight “PFW” (g), **G** plant dry weight “PDW” (g), **H** plant height “Ph” (cm), and **I** chlorophyll content (SPAD Unit) as influenced by six salt stress protectants (SP**)**, 15 ppm cobalt (Co1), 30 ppm (Co2), *B. subtilis* (Bs), (Co1 + Bs), (Co2 + Bs), and distilled water as a control (Cont.) and four levels of NaCl irrigation water salinity (SA); tap water as a control S0 = 0.5, S1 = 1.5, S2 = 4, and S3 = 6 dS m.^−1^. Bars with the same lowercase letters are not significantly different at the *P* < 0.05 level. The data are presented as the mean ± SE. The statistics are provided in additional file 1: Table S1

The means, of both seasons (2022 and 2023) for the NL, LL, SFW, RFW, RDW, PFW, PDW, Ph, and chlorophyll content of *Coriandrum sativum* L. cv. Balady, are presented in Additional file 1: Table S1 and (Fig. 2A, B, C, D, E, F, G, H, and I) (for details of two seasons, each in Additional file 2: Table S1). Among the studied coriander growth parameters, the chlorophyll content and morphology significantly differed when the salt stress level increased to S3 (6 dS m^−1^), as illustrated in Fig. 1a. Similarly, the mean values of the two seasons decreased by approximately 38, 37, 55, 37, 22, 55, 44, 26, and 12% for the NL, LL, SFW, RFW, RDW, PFW, PDW, Ph, and chlorophyll content, respectively.

Despite the salt stress, the cobalt and *B. subtilis* treatments had significant effects on all the studied coriander economic growth parameters (Fig. 1b, c, d and e, and Fig. 2A, B, C, D, E, F, G, H, and I), where the mean values of the coriander economic growth metrics were found to be the highest (Co2 + Bs), which increased the NL, LL, SFW, RFW, RDW, PFW, PDW, Ph, and chlorophyll content by approximately 30, 21, 28, 17, 18, 28, 29, 7, and 6%, respectively, compared with those of the control. Co1 + Bs had the second most significant effect after Co2 + Bs treatment on the mean average of all the economic growth parameters of the coriander in the two seasons, as shown in Additional file 1: Table S1 (Fig. 2A, B, C, D, E, F, G, H, and I). However, the lowest mean values in both seasons were achieved with the control treatment.

In general, different salt stress protectants had significant positive effects on the means of all the parameters, NL, LL, SFW, RFW, RDW, PFW, PDW, Ph, and chlorophyll content of the coriander plants in both seasons, the interactions between cobalt at 30 and 15 ppm, and *B. subtilis* in irrigation water produced the best mean values (NL 17, LL 22 cm, SFW 14 g, RFW 5 g, RDW 0.8 g, PFW 44 g, PDW 5 g, Ph 88 cm, and 44 SPAD). All the treatments increased the growth metrics of the stressed and non-stressed plants compared to those of the control plants (Additional file 1: Table S1) (Fig. 1b to e and Fig. 2A, B, C, D, E, F, G, H, and I).

### Elemental analysis and electrolyte leakage

**Fig. 3:**
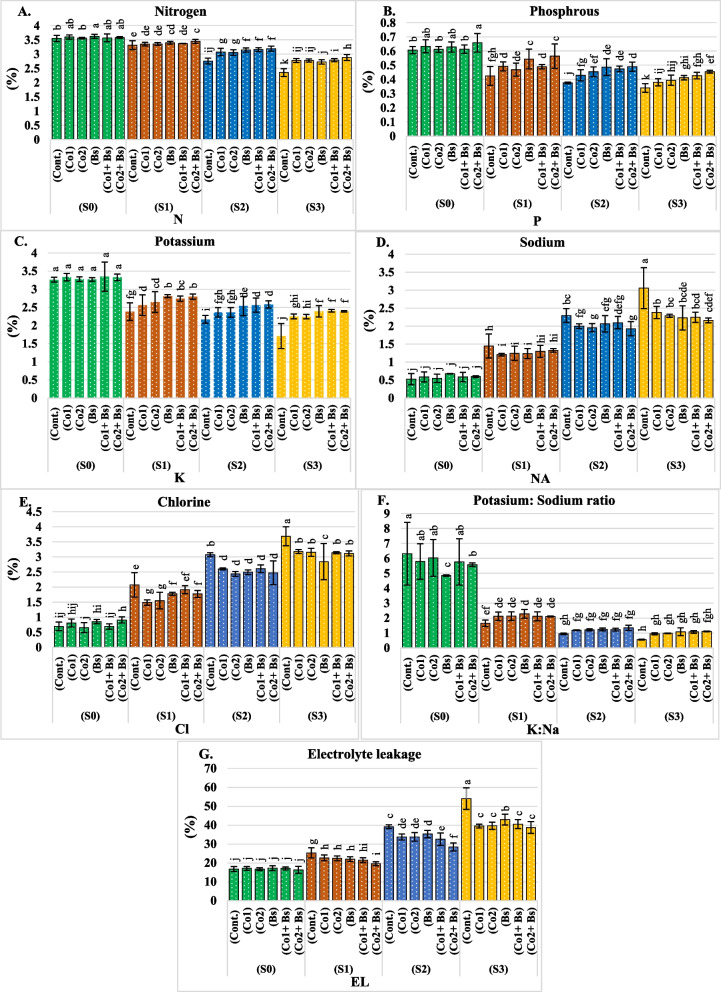
Elemental contents; **A** nitrogen “N” (%), **B** phosphorus “P” (%), **C** potassium “K” (%), **D** sodium “Na” (%), **E** chlorine “Cl” (%), **F** K:Na ratio, and **G** electrolyte leakage “EL” (%) of dry leaves in *Coriandrum sativum* L. cv. Balady as influenced by six salt stress protectants (SP**)**, 15 ppm cobalt (Co1), 30 ppm (Co2), *B. subtilis* (Bs), (Co1 + Bs), (Co2 + Bs), and distilled water as a control (Cont.) and four levels of NaCl irrigation water salinity (SA); tap water as a control S0 = 0.5, S1 = 1.5, S2 = 4, and S3 = 6 dS m.^−1^. Bars with the same lowercase letters are not significantly different at the *P* < 0.05 level. The data are presented as the mean ± SE. The statistics are provided in additional file 1: Table S2

In general, Additional file 1: Table S2 and (Fig. 3A, B, C, D, E, F, and G) present the means of nitrogen, phosphorus, potassium, sodium, and chlorine; the K:Na ratio; and electrolyte leakage of dry leaves in coriander plants for both seasons (2022 and 2023) (for details of two seasons, each alone in Additional file 2: Table S2). The leaf element content of Coriander plants was modulated by irrigation water salinity stress, salt stress protection, and their interaction.

There were correlations between increased NaCl concentrations and increasing Na^+^, Cl^−^, and electrolyte leakage levels. The leaf contents of Na^+^ and Cl^−^ and electrolyte leakage increased with increasing NaCl concentration (Fig. 3D, E, and G). However, there was a definite inverse relationship between the N, P, and K^+^ contents and between the K:Na ratio and NaCl concentration (Fig. 3A, B, C, and F) (Additional file 1: Table S2). Separated or combined cobalt and *Bacillus subtilis* treatments significantly reduced the Na + and Cl- levels as well as electrolyte leakage in coriander leaves (Fig. 3D, E, and G). Compared to those of the control, the other treatments had significant influences on increasing the N, P, and K + contents (Fig. 3A, B, and C) and (Additional file 1: Table S2).

Overall, the use of various SP increased the N, P, and K + contents and the K:Na ratio in coriander leaves grown under SA at the S1, S2, or S3 levels compared to those grown under the S0 level in both seasons. S0, S1, S2, and S3, (Co2 + Bs) had the highest mean N, P, and K^+^ contents and K:Na ratio, followed by (Co1 + Bs). Conversely, the lowest means were observed in the (Cont.) with S3, followed by S2, S1, and S0 (Fig. 3A, B, C, and F) and (Additional file 1: Table S2). The lowest means of Na^+^, Cl^−^, and electrolyte leakage in dry leaves of coriander plants in both seasons were recorded for Co2 + Bs, followed by Co1 + Bs under S0, S1, S2, and S3, respectively (Fig. 3D, E, and G) and (Additional file 1: Table S2).

### Antioxidant enzymes, Asco, and proline

**Fig. 4:**
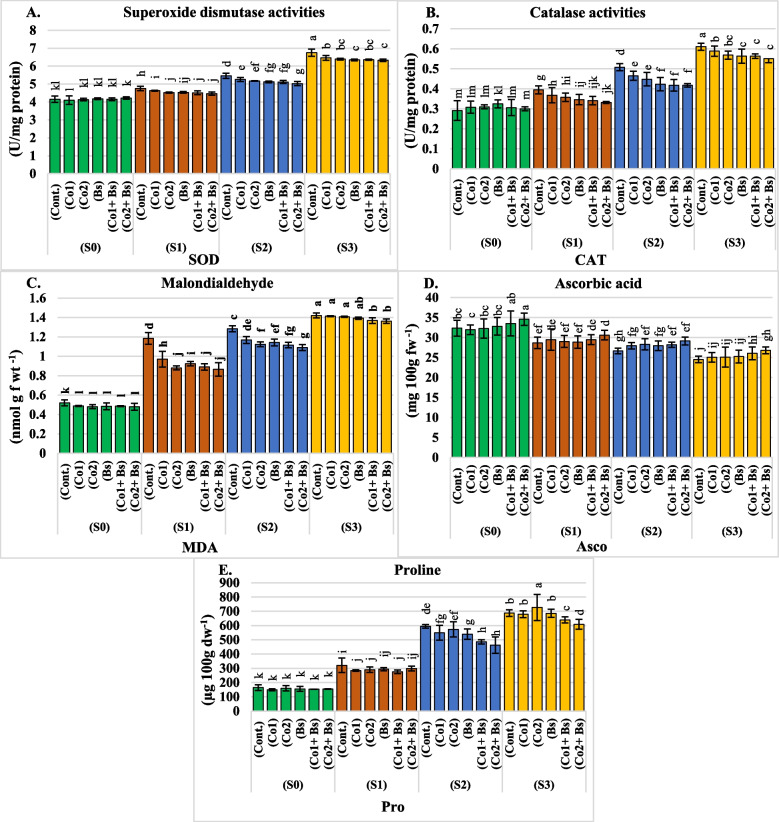
Determination of the activities of antioxidant enzymes: **A** superoxide dismutase “SOD” (U/mg protein); **B** catalase “CAT” (U/mg protein); **C** malondialdehyde “MDA” (nmol g f wt^−1^); **D** ascorbic acid “Asco” (mg 100 g f wt^−1^); and **E** proline “Pro” (µg 100 g d wt^−1^) content in the leaves of *Coriandrum sativum* L. cv. Balady, as influenced by six salt stress protectants (SP**)**, 15 ppm cobalt (Co1), 30 ppm (Co2), *B. subtilis* (Bs), (Co1 + Bs), (Co2 + Bs), and distilled water as a control (Cont.) and four levels of NaCl irrigation water salinity (SA); tap water as a control S0 = 0.5, S1 = 1.5, S2 = 4, and S3 = 6 dS m.^−1^. Bars with the same lowercase letters are not significantly different at the *P* < 0.05 level. The data are presented as the mean ± SE. The statistics are provided in additional file 1: Table S3

Our results showed that irrigation water salinity, salt stress protection agent application, and their interaction significantly (*P* < 0.05) affected the activities of the antioxidative enzymes SOD, CAT, and MDA; Asco; and proline in coriander leaves (Additional file 1: Table S3) and (Fig. 4A, B, C, D, and E) (for details of two seasons, each alone in Additional file 2: Table S3). Similarly, the activities of SOD, CAT, MDA, and proline increased significantly when the salt stress level increased to S3 (Fig. 4A, B, C, and E). Conversely, when the Asco decreased significantly (Fig. 4D).

The combination of Co2 with Bs had significant effects on reducing SOD, CAT, MDA and proline levels, with mean values of 5.0, 0.4, 0.94 (mg 100 g f wt^−1^), and 380.70 (µg 100 g d wt^−1^), respectively, compared to those of the control, with mean values of 5.27, 0.45, 1.10 (mg 100 g f wt^−1^), and 441.77 (µg 100 g d wt^−1^), for both seasons (Additional file 1: Table S3) and (Fig. 4A, B, C, and E). In contrast, the Asco increased significantly (Fig. 4D) to 30.28 (mg 100 g f wt^−1^). In addition, Co1 or Co2 in combination with Bs had a decrease in SOD, CAT, and MDA activity and proline levels, but (Co2 + Bs) had the greatest reduction in both S2 and S3 (Additional file 1: Table S3) (Fig. 4A, B, C, and E). Compared to those in the control treatment, the Co2 + Bs treatment had the most significant impact on increasing Asco in S2 and S3 (Additional file 1: Table S3) (Fig. 4D).

### Important phytochemicals in the seed oil of coriander

**Fig. 5:**
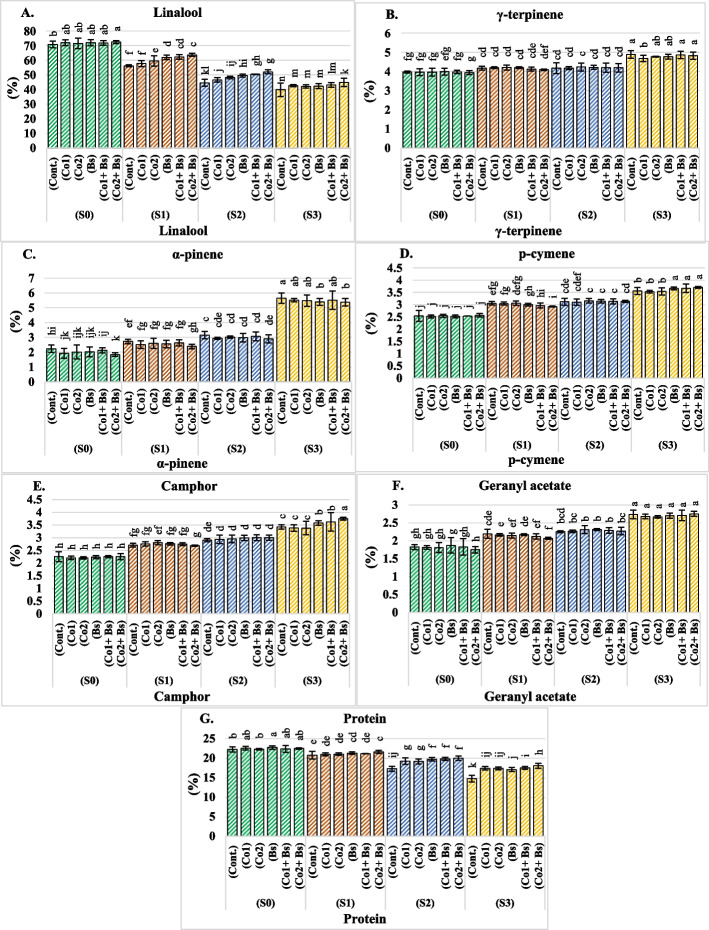
Important biological phytochemicals related to the seed oil of *Coriandrum sativum* L. cv. Balady, **A** linalool (%), **B** γ-terpinene (%), **C** α-pinene (%), **D** p-cymene (%), **E** camphor (%), **F** geranyl acetate (%), and **G** protein (%) influenced by six salt stress protectants (SP**)**, 15 ppm cobalt (Co1), 30 ppm (Co2), *B. subtilis* (Bs), (Co1 + Bs), (Co2 + Bs), and distilled water as a control (Cont.) and four levels of NaCl irrigation water salinity (SA); tap water as a control S0 = 0.5, S1 = 1.5, S2 = 4, and S3 = 6 dS m.^−1^. Bars with the same lowercase letters are not significantly different at the *P* < 0.05 level. The data are presented as the mean ± SE. The statistics are provided in additional file 1: Table S5

The means of both seasons (2022 and 2023) for the important biological phytochemicals linalool, γ-terpinene, α-pinene, p-cymene, camphor, geranyl acetate, and protein of coriander are illustrated in Additional file 1: Table S4 and Fig. 5A, B, C, D, E, F, and G (for details of two seasons, each alone is shown in Additional file 2: Table S4). The findings revealed that the phytochemicals present in the oil varied depending on the treatment and their interactions. The levels of γ-terpinene, α-pinene, p-cymene, camphor, and geranyl acetate significantly increased when the amount of salt stress increased to S3 (Fig. 5B, C, D, E, and F). However, the linalool and protein levels decreased significantly (Fig. 5A and G).

Cobalt and *B. subtilis* treatments alone or in combination had significant influences on the increase in linalool, camphor, and protein levels relative to those in the control (Fig. 5A, E, and G). However, the same tendency was not observed for the levels of γ-terpinene and p-cymene, as no significant differences were detected for their corresponding (cont.) (Fig. 5B and D). However, the levels of α-pinene and geranyl acetate decreased significantly (Fig. 5C and F). The varied concentrations of Co with Bs reversed the negative effect of salinity, causing a reduction in linalool, camphor, and protein levels compared with those of the stressed plants without such application (Cont.) (Fig. 5A, E, and G). At varying levels of salt stress, the lowest reductions in linalool, camphor, and protein levels were found in the Co2 + Bs treatment group. Furthermore, Co1 performed similarly to the other treatments in terms of reducing the amounts of α-pinene and geranyl acetate under varying levels of salt stress (Fig. 5C and F).

### Coriander yield parameters of seeds and oil

**Fig. 6:**
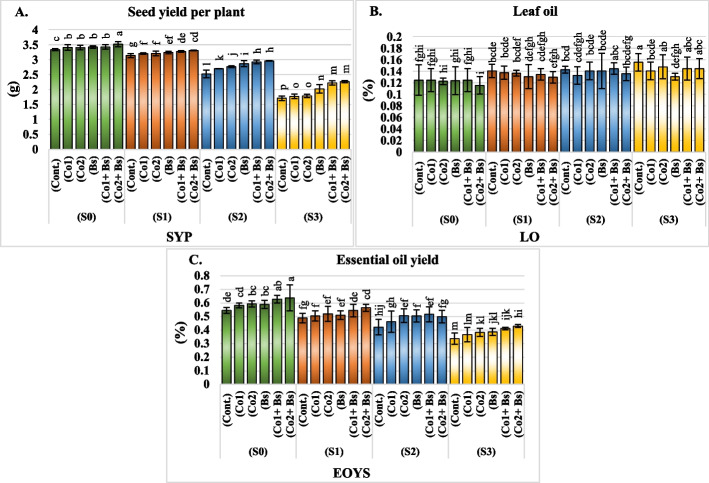
Yield parameters of *Coriandrum sativum* L. cv. Balady seed and oil; **A** seed yield per plant “SYP” (g), **B** leaf oil “LO” (%), and **C** essential oil yield “EOYS” influenced by six salt stress protectants (SP**)**, 15 ppm cobalt (Co1), 30 ppm (Co2), *B. subtilis* (Bs), (Co1 + Bs), (Co2 + Bs), and distilled water as a control (Cont.) and four levels of NaCl irrigation water salinity (SA); tap water as a control S0 = 0.5, S1 = 1.5, S2 = 4, and S3 = 6 dS m.^−1^. Bars with the same lowercase letters are not significantly different at the *P* < 0.05 level. The data are presented as the mean ± SE. The statistics are provided in additional file 1: Table S3

An increase in salinity to the greatest concentration in both seasons significantly reduced the weight of the seed yield per plant and the percentage of essential oil yield of the coriander plants by approximately 42% and 35%, respectively. However, the leaf oil percentage significantly increased with increasing salinity up to the highest concentration, reaching approximately 14% in both seasons under the same amount of salt stress (Additional file 1: Table S5) and (Fig. 6A, B, and C) (for details of the two seasons, each alone in Additional file 2: Table S5).

The combination or combination of Co and Bs had a significantly positive effect on both the weight of the seed yield per plant and the percentage of essential oil yield of the coriander plants, regardless of salt stress. However, both the Co2 and (Co1 + Bs) treatments had the same positive effect on preventing the leaf oil percentage from decreasing. The interaction effect between salinity and salt stress protection agent treatment had significant effects on the coriander yield, percentage of essential oil yield, and leaf oil percentage (Additional file 1: Table S5) (Fig. 6A, B, and C).

Under low, moderate, and high salinity (S1, S2, and S3), (Co2 + Bs) exhibited the greatest improvement, but at the same level, in terms of seed yield per plant and percentage of essential oil yield but not in leaf oil percentage. The highest seed yield per plant and the percentage of essential oil yield were obtained at (Co2 + Bs), followed by (Co1 + Bs) for the Coriander plants even with the high salinity (S3). However, at the same salt level, the Co2 treatments produced equivalent results in terms of leaf oil percentage after (Cont.) Under the unstressed salt treatment (S0) and (Co2 + Bs), the highest values of seed yield per plant and percentage of essential oil yield were observed, followed by the values in (Co1 + Bs) when compared with the corresponding values (Cont.), Additional file 1: Table S5 and Fig. 6A, B, and C (for details of two seasons, each alone in Additional file 2: Table S5).

## Discussion

Our results revealed significant negative responses of all the recorded growth parameters, as well as morphological, biochemical, and physiological processes, in coriander plants exposed to NaCl salt stress, which is more harmful to plants than other salt-induced salinities. The same results were reported by Dustgeer et al. [52] and Sadak et al. [53] as they mentioned salt stress inhibits plant growth quality and productivity in maize and white lupine in a variety of ways, such as ionic and osmotic imbalance; a lack of water and nutrient availability, such as N, P, and K; decreased photosynthesis pigment synthesis; and disturbance of photosynthetic activity, plant cells experienced an instant response to salt stress, while osmotic stress was brought on by ions surrounding roots that inhibited their ability to absorb water, which causes limited growth. The number of leaves, leaf length, shoot fresh weight, root fresh weight, root dry weight, plant fresh weight, plant dry weight, plant height, and SPAD index were significantly lower as the salinity concentration increased in coriander [7, 49] and *Moringa* [4]; these decreases in all growth parameters under salt stress could be attributed to thylakoid enlargement, ion accumulation, and functional abnormalities observed during stoma closure and opening, as well as harm to the photosynthetic machinery and chloroplast structure, reactive oxygen species under salinity stress primarily target chloroplasts, leading to the destruction of photosynthetic pigments and thylakoid membranes. Increased salt ion accumulation not only impaired morphological growth but also had a significant negative response to the percentage of N, P, and K and the K:Na ratio in coriander leaves; earlier research supported similar findings in coriander plants [49, 54]; this decrease may be due to the low osmotic potential of the soil solution, overaccumulation of Na and Cl in the cells, and competition with Na, resulting in a significant positive response in the percentage of electrolyte leakage [55]. Recent findings have shown that increased salt ion accumulation causes an increase in antioxidant defense mechanisms, such as superoxide dismutase (SOD), catalase (CAT), and malondialdehyde (MDA), and the accumulation of osmolytes, such as proline, in all plant cells to maintain turgor pressure, which is vital for cell expansion. However, Asco was reduced, several authors have noted this occurrence [56–58] in wheat and Hassanein et al. 2022 [59] in coriander. According to these investigations, one of the reasons why salinized coriander plants were unable to tolerate salt stress was a decrease in Asco. Ascorbic acid is a tiny, water-soluble antioxidant that plays an important role in the cyclic pathway of H_2_O_2_ enzymatic detoxification. Furthermore, increased amino acid generation, particularly proline generation, is indirectly associated with the scavenging of oxidative stress and prevents cellular damage by stimulating the production of several antioxidant enzymes, such as SOD, CAT, and MDA, which are vital for osmotic stress resistance in plants under abiotic stress [60–63]. Acimovic et al. [64] studied coriander plants and discovered that increasing the concentration of bio compounds such as γ-terpinene, α-pinene, p-cymene, camphor, and geranyl acetate in fruit essential oils had a significant response to abiotic stress, such as salinity, at the maximum NaCl concentration. These results were found to be in line with the current results. On the other hand, protein levels decreased, which may be attributed to the rate of biosynthesis and degradation associated with N, P, and K accumulation, which decreased as salt ion levels increased because these are important nutrients that play a major role in protein synthesis and metabolism in coriander plants. Additionally, reduced linalool levels are correlated with increased salt stress and abiotic stress [64, 65]; these findings are consistent with the present study. Our results illustrated that salt stress adversely affects the seed yield per plant (SYP) and essential oil yield (EOYS %), possibly due to limited macro and micronutrient availability; in particular, the adsorption of N, P, and K under increased salt stress causes a decrease in the biosynthesis of amino acids, which subsequently negatively affects the growth rate; the relative content of leaf water, protein, and chlorophyll contents in coriander plants; the exchange of gases with the net CO_2_ acceptance rate; the photosynthesis efficiency; the transpiration rate, all of which are required for flowering and pollination; and even the eventual transfer of assimilates and filling of seeds (SYP and EOYS) [66–68]. In contrast, when LO levels increase, a greater LO quantity under saline conditions could be connected to an increase in the number and density of oil glands [69].

In this study, the preventative effects of Bs and Co on coriander plants exposed to salt stress were investigated and compared. When coriander plants growing under salt stress were treated with Co or Bs, whether together or separately, our results revealed a significant positive response of all the recorded growth parameters; the number of leaves, leaf length, shoot fresh weight, root fresh weight, root dry weight, plant fresh weight, plant dry weight, plant height, and SPAD index when compared to those of nontreated plants; however, the concentration of Co applied to the plant also played an important role. Several studies have shown that some halophilic *Bacillus* species, as dominant bacterial genera under salt stress, act by externally protecting the cell wall, endospore-forming capacity, healthy cell membrane, and enzymatic mechanisms to handle and regulate environmental dangers. Furthermore, Bs exhibit a variety of metabolic and adaptable reactions to varied water supplies, water consumption efficiencies, available N, P, K oxygen, and organic/inorganic materials [70] in *Capsicum* [71]*.* Species such as *B. subtilis* potentially minimize soil N loss [72]; accelerate N uptake and transport [73]; and produce indole-3-acetic acid (IAA), cytokinins, siderophores, and gibberellins, which tend to promote plant growth characteristics by encouraging significant cell division, proliferation, and differentiation in the meristematic area; light and gravity responses; root and shoot initiation, development and elongation; vegetative growth; and yield development [74, 75]. Along with the endogenous IAA content, bacterial IAA loosens plant cell walls; induces cell division in plants; leads to the production of ACC deaminase; decreases plant ethylene levels; and changes in root form and development; increases surface area, volume, and length; and promotes indole-mediated signaling [76] in *Withania somnifera*, which allows the plant to obtain nutrients effectively under salt stress conditions and C-N metabolism control; and improves the conversion of inorganic N to amino acids, resulting in significant plant growth stimulation [77–80]. Tryptophan is the primary precursor of bacterial indole chemical products detected in root secretions, and it enhances protein synthesis and RNA polymerase activity [81, 82]. Jie et al. [83], preserve ionic equilibrium in plants under salt stress by enhancing the preferential absorption of K over Na into stressed plant root xylem; increasing or regulating K + /Na + ratios; and increasing phosphorus and iron availability, all of which significantly increase plant growth parameters, fresh root weight, dry root weight in blackberry plants [84], yield, and quality of several medicinal plants [85, 86]. Vardharajula et al. [87] studied *Zea mays*, Chun et al. [88] tomatoes, Yasmeen et al. [89] sunflower plants, Medeiros and Bettiol [90] reported that Bs not only increased amino acid and carbohydrate uptake and buildup, which may act as essential N and/or C suppliers for increased production of proteins and peptides but also reduced the antioxidant activity of enzymes, such as CAT, SOD, and MDA, in *Camellia sinensis* and quinoa [72, 84]. However, as the Asco concentration increased, the Asco concentration significantly influenced the defense system of the plants. Additionally, Bs significantly reduced proline buildup and electrolyte leakage; these findings are consistent with our present findings. Consequently, proteins trigger protoplasm and vegetative growth, and more nitrogen is provided. Moreover, a plant with several leaves can produce a significant amount of seeds and essential oils [91] in *Coriandrum sativum* [92] *Foeniculum vulgare* [93] and chamomile plants as demonstrated by the high fresh and dry weight, and height [70, 94]. Furthermore, phosphate-solubilizing bacteria enhance grain yield, herbage and oil yield, chlorophyll, and photosynthetic leaf area but decrease the number, diameter, and distribution of glandular hairs per leaf where oil is produced and preserved, resulting in a decrease in leaf oil in *Mentha piperita* medicinal plants under stress [95]. Bacterial growth regulators may control the majority of metabolic and physiological activities, including metabolic rates and essential oil synthesis, in medicinal and aromatic plants. Moreover, increasing concentrations of essential oil can increase the quantity of essential oil [96] in *Ocimum* [97] in *Salvia* and [98] in ajwain and change the composition of essential oil, impacting the process of terpenoid biosynthesis to generate a stress factor that triggers defense mechanisms [74]. Secondary metabolite regulation or accumulation and flavonoid, phenolic, and hydrogen peroxide accumulation in plants are indicators of healthy adaptation to stress, as these compounds coordinate osmotic balance, preserve cytoplasmic macromolecules from water loss, and act as free radical scavengers [99]. Our results revealed that Bs significantly increased the levels of major essential oil components, which is consistent with the findings of several studies; for example, the levels of linalool in *Mentha piperita* [100] and *Ocimum basilicum* [101]; the contents of camphor in *Salvia officinalis* [102]; and the contents of geranyl acetate [103], the increase in essential oil components may be correlated with macronutrients; N, P, K availability; uptake [104]; and plant cells [105] in *Mentha piperita* [106] in basil, which fix free atmospheric nitrogen through leaves and roots [31, 107]. However, the amount of α-pinene fluctuates toward lower concentrations and has no significant effect on the levels of γ-terpinene or p-cymene in *O. syriacum* [108].

Our findings revealed that the level of Co had a significant impact on improving the NL, LL, SFW, PFW, RFW, RDW, PDW, Ph, and chlorophyll content during coriander plant growth compared with those of the control, and treatment with 30 ppm Co (Co2) outperformed treatment with 15 ppm Co (Co1). This difference may be attributed to the increase in root N, P, and K^+^ uptake; increase in K^+^ content; and decrease in Na, Cl, and electrolyte leakage in leaves, which were also observed in our study and are important for coriander plant resistance to salt stress. Recent studies have additionally demonstrated that the effect of Co on growth is connected to a rise in the macronutrient levels of stressed plants in Bhendi [109], stimulating hormone synthesis (increased expression of auxins and gibberellins) and decreasing the activity of several enzymes (catalase and peroxidase), thus enhancing anabolism and reducing catabolism [18, 110]. Co is an essential micronutrient for vitamin B_12_ [17] and is necessary for various enzymes and proteins and regulates N2 fixation and cell balance through interactions with other critical micronutrients, such as iron (Fe), nickel (Ni), and zinc (Zn), in plant metabolism [24, 111]. Co significantly impacts methionine synthesis, which in turn promotes protein synthesis, ribonucleotide reductase is a vitamin B_12_-dependent enzyme that converts ribonucleotides to deoxyribonucleotides, a process that reduces the rate of DNA synthesis [112]. Peroxidases are isoenzymes that catalyze redox reactions, including the breakdown of hydrogen peroxide. Han et al. [113] discovered that Co affects peroxidase efficiency by binding to certain amino acids near the enzyme's active regions in horseradish. Co reduces the activity of the antioxidant enzymes SOD, CAT, and MDA. This difference may be responsible for the decrease in ROS levels resulting from CO application [114, 115]. Its application to coriander plants enhances the levels of nonenzymatic antioxidants such as Asco and Pro in response to increasing Co concentrations [116], which are also useful for decreasing injury to the cell membrane via oxidative stress because of their antioxidative activity in plants [117]. Co treatments have been shown to increase coriander herb yield; mineral and phytochemical contents; and seed oil constituents, such as linalool, SYP, LO, and EOYS, while also preserving γ-terpinene, p-cymene, camphor, and geranyl acetate compared to those of control plants [118]. This could be due to numerous stimulating effects on hormonal synthesis and metabolism, which are ascribed to catalase and peroxidase activities that improve all development indices of Lemongrass (*Cymbopogon citratus*) [119] and peppermint [120], these results were confirmed to be consistent with the existing results.

Salt stress inhibits plant root growth and development, leading to reduced nutrient uptake. We noticed that, compared with the other treatments of eco-safe salt stress protection agents, the interaction of Co at 30 ppm with Bs had synergistic effects on most of the growth parameters and characteristics of coriander plants, particularly those under significant salt level stress. Several studies have shown that cobalt [CoSO_4_.7 H_2_O] and *Bacillus subtilis* play independent roles [121]. The beneficial effects of the combined treatment could be attributed to the fact that particular bacteria demand submicromolar levels of cobalt for growth and have specific systems for cobalt absorption, release, and transcriptional modification that preserve cobalt homeostasis [122]. Adding cobalt to the *Bacillus* environment increases the capacity of bacteria to create extracellular alkaline phosphatase [123]. The concentration of cobalt is associated with its ability to promote the synthesis of alkaline phosphatase, which accounts for a large portion of all proteins produced during the late logarithmic and initial stationary stages of development [124]. However, the effect of cobalt in combination with *Bacillus subtilis* on optimizing the productivity of medicinal and aromatic plants under salt stress has rarely been studied. Taken together, the results of the present study showed the combined effects of bacteria and cobalt, especially the combined effects of Co2 + Bs, and the optimal results may be attributed to the rapid release of available nitrogen synthesized by root rhizobia to the plant during vegetative growth; high soil nitrogen fixation; rapid availability of N, P, and K in the rhizosphere; decreased aggregation and absorption of Na^+^ and Cl^−^; and osmotic correction of the Na^+^/K^+^ ratio, which increases photosynthesis pigments, such as total chlorophyll, NL, LL, SFW, RFW, RDW, PFW, PDW, Ph, and chlorophyll content, from improved nodulation; the same results were reported previously [125, 126]. In addition, plant phytohormones, indole-3-acetic acid (IAA), cytokinins, siderophores, and gibberellins are modified and produced under environmental stress [127, 128], and protein synthesis and RNA polymerase activity are enhanced, encouraging significant cell division, proliferation, and differentiation in the meristematic area [129, 130]. cell balance through interactions with other critical micronutrients; decrease in ROS levels; CAT, SOD, MDA, and proline levels; and electrolyte leakage [71, 131]. The yield, quality, and therapeutic value of the coriander essential oil are based on its linalool, γ-terpinene, p-cymene, camphor, and protein contents. It is crucial for the cultivation of one of the most economically significant medicinal and aromatic plants, particularly when exposed to salt stress. Our results showed that Co and Bs, either together or individually, increase the amount of these compounds as well as yield. This difference may be related to the superior ability of these materials to reduce Na^+^ and Cl^−^ in coriander leaves. Shahid et al. [132] and Ha-Tran et al. [133] reported that reducing Na^+^ and Cl^−^ in plants significantly contributes to yield improvement under salinity stress.

## Conclusion

The protective effects of eco-safe Bs and Co under salt stress have been evaluated through comparisons of coriander plant herb growth, yield, essential oil productivity, and physiological and phytochemical responses. Our unique findings revealed that the combination of Bs with Co (30 ppm) can significantly minimize salt stress-induced damage and optimize the growth of coriander plants through the reduction of ROS-scavenging enzymes such as SOD, CAT, and MDA and non-enzyme such as proline while preserving ascorbic acid and vital nutrient levels and enhancing plant osmotic potential to buffer salt stress. Concurrently, the seed yield per plant, leaf oil yield, essential oil yield, and quality have been indicated by the high quantities of the vital biological phytochemicals linalool, γ-terpinene, camphor, geranyl acetate, and protein. Furthermore, the protective effect of the Bs treatment was greater than that of the Co treatment (30 ppm or 15 ppm) in lowering the Na^+^ and Cl^−^ contents, which may lead to greater total yield and quality under excessive salt stress. This investigation provides vital details regarding the role of Co in redox homeostasis and the involvement of Bs in optimizing the quality and quantity of coriander plant productivity under salt stress. Interestingly, further investigations are suggested to clarify the exact mechanism of action of the dual impact of Bs and Co on medicinal and aromatic plant salt stress tolerance.

## Supplementary Information

Additional file 1.

Additional file 2.

## Data Availability

This published paper and the supplementary data contain all the data created or analyzed during this investigation.
