# Supplementary material for: Promoting salt tolerance, growth, and phytochemical responses in coriander (Coriandrum sativum L. cv. Balady) via eco-friendly Bacillus subtilis and cobalt
Source: BMC Plant Biol. 2024 Sep 10;24:848. doi: 10.1186/s12870-024-05517-3 (PMC11384715; doi:10.1186/s12870-024-05517-3)
Supplement: Supplementary file 1 — Additional file 1. [file 12870_2024_5517_MOESM1_ESM.docx]

6 X 4 = 24 Treatments **Two seasons (together)**

**S1** Growth parameters and characteristics

1. Number of leaves “NL”
2. Leaf length “LL” (cm)
3. Shoot fresh weight “SFW” (g)
4. Root fresh weight “RFW” (g)
5. Root dry weight “RDW” (g)
6. Plant fresh weight “PFW” (g)
7. Plant dry weight “PDW” (g)
8. Plant height “Ph” (cm)
9. Chlorophyll content (SPAD Unit)

**Chemical measurements and analyses**

**S2** Elements content dry leaves

1. Nitrogen “N” (%)
2. Phosphorus “P” (%)
3. Potassium “K” (%)
4. Sodium “Na” (%)
5. Chlorine “Cl” (%)
6. K:Na ratio
7. Electrolyte leakage “EL” (%)

**S3** Determine the activity of antioxidant enzymes, Ascorbic acid, and Proline content in leaf 

1. Specific activity of superoxide dismutase “SOD” (U/mg protein)
2. Catalase activities “CAT” (U /mg protein)
3. Malondialdehyde “MDA” (nmol/g F.wt.) = MDA (nmol g f wt^-1^)
4. Ascorbic acid “Asco” (mg/100 g f. wt.)
5. Proline “Pro” (µg /100 g D. wt.)

**S4** Percentage of important biological phytochemicals in coriander seed oil

1. Linalool “Linalool” (%)
2. γ-terpinene “γ-terpinene” (%)
3. α-pinene “α-pinene” (%)
4. p-cymene “p-cymene” (%)
5. Camphor “Camphor” (%)
6. Geranyl acetate “Geranyl acetate” (%)
7. Protein (%)

**S5** Yield parameters of seed and oil

1. Seed yield per plant “SYP” (g)
2. Leaf oil “LO” (%)
3. Essential oil yield “EOYS” (%)

**Supplementary Tables S1.**

Influence of applied six eco-safe salt stress protectants (SP); cobalt (Co1) at 15 ppm, (Co2) at 30 ppm, *Bacillus subtilis* (Bs), (Co1 + Bs), (Co2 + Bs), and distilled water as a control (Cont.) to irrigation water under four levels of NaCl irrigation water salinity (SA); tap water as a control (S0; 0.5, S1; 1.5, S2; 4 and S3; 6 dS m^−1^) and their interaction on growth parameters and characteristics of *Coriandrum sativum* L. cv. Balady. Values are in the form of means of both seasons 2022 and 2023.

| Treatments | | NL | LL (cm) | SFW (g) | RFW (g) | RDW (g) | PFW (g) | PDW (g) | Ph (cm) | Chlorophyll (SPAD Unit) |
| --- | --- | --- | --- | --- | --- | --- | --- | --- | --- | --- |
| The main plot (SA) | (S0) | 16.027 A | 21.886 A | 13.879 A | 5.482 A | 0.846 A | 41.636 A | 5.412 A | 86.753 A | 43.722 A |
|  | (S1) | 14.250 B | 19.383 B | 11.315 B | 4.830 B | 0.794 B | 33.943 B | 4.718 B | 79.574 B | 41.555 B |
|  | (S2) | 13.861 C | 17.913 C | 8.095 C | 3.986 C | 0.714 C | 24.442 C | 3.608 C | 70.707 C | 40.416 C |
|  | (S3) | 9.972 D | 13.986 D | 6.283 D | 3.488 D | 0.660 D | 18.847 D | 3.031 D | 64.647 D | 38.722 D |
| Main effect of subplot (SP) | (Cont.) | 10.416 E | 15.191 C | 7.921 D | 3.973 D | 0.670 D | 23.761 D | 3.326 D | 71.566 C | 39.416 C |
|  | (Co1) | 12.458 D | 18.433 B | 9.507 C | 4.383 C | 0.742 C | 28.522 C | 4.028 C | 75.635 B | 41.125 B |
|  | (Co2) | 13.541 C | 19.237 A | 10.457 B | 4.422 C | 0.750 C | 31.012 B | 4.381 B | 77.021 A | 41.583 AB |
|  | (Bs) | 14.291 B | 18.566 AB | 10.147 B | 4.459 C | 0.757 C | 30.440 B | 4.299 B | 75.275 B | 41.250 B |
|  | (Co1+ Bs) | 15.541 A | 19.079 AB | 10.399 B | 4.599 B | 0.779 B | 31.198 B | 4.403 B | 75.487 B | 41.250 B |
|  | (Co2+ Bs) | 14.916 AB | 19.245 A | 11.123 A | 4.843 A | 0.823 A | 33.371 A | 4.717 A | 77.537 A | 42.000 A |
| The interaction between (SA × SP) | | | | | | | | | | |
| (S0) | (Cont.) | 14.833 c-e | 20.783 b-d | 12.386 fg | 5.204 cd | 0.804 c-e | 37.158 fg | 4.830 ef | 83.615 c | 43.166 b |
|  | (Co1) | 14.833 c-e | 22.200 ab | 13.350 de | 5.372 bc | 0.830 b-d | 40.048 de | 5.206 cd | 86.473 b | 44.500 a |
|  | (Co2) | 15.666 bc | 22.866 a | 13.791 cd | 5.373 bc | 0.829 b-d | 41.371 cd | 5.378 bc | 87.393 b | 43.333 ab |
|  | (Bs) | 16.666 ab | 21.433 a-c | 14.270 bc | 5.416 b | 0.836 bc | 42.806 bc | 5.564 ab | 86.430 b | 43.166 b |
|  | (Co1+ Bs) | 16.833 ab | 21.616 a-c | 14.543 ab | 5.536 b | 0.854 b | 43.630 ab | 5.672 a | 87.553 ab | 43.666 ab |
|  | (Co2+ Bs) | 17.333 a | 22.416 a | 14.935 a | 5.993 a | 0.927 a | 44.803 a | 5.824 a | 89.053 a | 44.500 a |
| (S1) | (Cont.) | 11.833 g | 15.833 h | 9.518 k | 4.237 g | 0.691 g-i | 28.550 k | 3.969 hi | 75.865 e | 40.333 e-g |
|  | (Co1) | 13.333 f | 19.250 e-g | 10.683 j | 4.896 e | 0.803 c-e | 32.050 j | 4.455 g | 80.501 d | 40.333 e-g |
|  | (Co2) | 14.333 d-f | 20.183 c-e | 12.050 gh | 4.860 e | 0.799 de | 36.146 gh | 5.024 de | 80.401 d | 41.833 cd |
|  | (Bs) | 15.000 cd | 19.716 d-f | 11.270 ij | 4.944 e | 0.814 c-e | 33.810 ij | 4.698 fg | 79.525 d | 42.500 bc |
|  | (Co1+ Bs) | 16.500 ab | 20.866 b-d | 11.636 hi | 4.996 e | 0.823 b-d | 34.911 hi | 4.851 ef | 80.346 d | 41.833 cd |
|  | (Co2+ Bs) | 14.500 c-f | 20.450 c-e | 12.731 ef | 5.050 de | 0.832 b-d | 38.195 ef | 5.308 bc | 80.806 d | 42.500 bc |
| (S2) | (Cont.) | 9.333 i | 13.65 i | 5.876 o | 3.413 k | 0.612 k | 17.631 o | 2.607 l | 67.260 h | 38.000 i |
|  | (Co1) | 12.000 g | 18.016 g | 7.471 m | 3.760 ij | 0.673 h-j | 22.413 m | 3.308 k | 70.096 g | 40.166 d-f |
|  | (Co2) | 13.666 ef | 18.966 e-g | 8.847 kl | 3.899 hi | 0.699 gh | 26.720 kl | 3.941 hi | 72.901 f | 41.500 c-e |
|  | (Bs) | 14.166 d-f | 18.600 fg | 8.390 l | 4.003 h | 0.719 fg | 25.170 l | 3.719 ij | 71.063 g | 40.333 e-g |
|  | (Co1+ Bs) | 17.166 a | 19.100 e-g | 8.988 kl | 4.397 fg | 0.787 e | 26.968 kl | 3.979 hi | 70.126 g | 41.333 c-f |
|  | (Co2+ Bs) | 16.833 ab | 19.150 e-g | 9.250 k | 4.446 f | 0.797 de | 27.753 k | 4.093 h | 72.796 f | 41.166 f-h |
| (S3) | (Cont.) | 5.666 j | 10.500 j | 3.903 p | 3.040 l | 0.575 l | 11.705 p | 1.899 m | 59.525 j | 36.166 j |
|  | (Co1) | 9.666 i | 14.266 i | 6.526 n | 3.505 k | 0.663 ij | 19.580 n | 3.142 k | 65.470 i | 39.500 gh |
|  | (Co2) | 10.500 hi | 14.933 hi | 6.603 n | 3.557 jk | 0.673 h-j | 19.810 n | 3.182 k | 67.390 h | 39.666 gh |
|  | (Bs) | 11.333 gh | 14.516 hi | 6.660 n | 3.474 k | 0.657 ij | 19.975 n | 3.214 k | 64.081 i | 39.000 hi |
|  | (Co1+ Bs) | 11.666 gh | 14.733 hi | 6.428 no | 3.467 k | 0.653 j | 19.283 no | 3.111 k | 63.923 i | 38.166 i |
|  | (Co2+ Bs) | 11.000 gh | 14.966 hi | 7.578 m | 3.884 hi | 0.736 f | 22.733 m | 3.641 j | 67.495 h | 39.833 gh |
| LSD_0.05_ (SA) | | 0.371 | 0.740 | 0.276 | 0.065 | 0.011 | 0.797 | 0.106 | 0.435 | 0.554 |
| LSD_0.05_ (SP) | | 0.652 | 0.757 | 0.336 | 0.103 | 0.0178 | 0.954 | 0.134 | 0.815 | 0.653 |
| LSD_0.05_ (SA × SP) | | 1.304 | 1.515 | 0.788 | 0.207 | 0.035 | 1.909 | 0.269 | 1.631 | 1.306 |

LSD_0.05_ = least significant differences at 0.05 probability. Means with the same letters in the same column are not significantly different (P ≤ 0.05) according to Tukey's test. Number of leaves “NL”, Leaf length “LL” (cm), shoot fresh weight “SFW” (g), root fresh weight “RFW” (g), Root dry weight “RDW” (g), plant fresh weight “PFW” (g), plant dry weight “PDW” (g), plant height “Ph” (cm), and Chlorophyll content (SPAD Unite).

**Supplementary Tables S2.**

Influence of applied six salt stress protectants (SP); cobalt (Co1) at 15 ppm, (Co2) at 30 ppm, *Bacillus subtilis* (Bs), (Co1 + Bs), (Co2 + Bs), and distilled water as a control (Cont.) to irrigation water under four levels of NaCl irrigation water salinity (SA); tap water as a control (S0; 0.5, S1; 1.5, S2; 4 and S3; 6 dS m^−1^) and their interaction on elements content, K:Na ratio and Electrolyte leakage of dry leaves in *Coriandrum sativum* L. cv. Balady. Values are in the form of means of both seasons 2022 and 2023.

| Treatments | | N (%) | P (%) | K (%) | Na (%) | Cl (%) | K:Na ratio | EL (%) |
| --- | --- | --- | --- | --- | --- | --- | --- | --- |
| The main plot (SA) | (S0) | 3.586 A | 0.626 A | 3.306 A | 0.588 D | 0.771 D | 5.719 A | 16.842 D |
|  | (S1) | 3.379 B | 0.498 B | 2.658 B | 1.295 C | 1.764 C | 2.070 B | 22.222 C |
|  | (S2) | 3.068 C | 0.452 C | 2.431 C | 2.058 B | 2.616 B | 1.192 C | 33.802 B |
|  | (S3) | 2.721 D | 0.402 D | 2.234 D | 2.395 A | 3.188 A | 0.96 D | 42.560 A |
| Main effect of subplot (SP) | (Cont.) | 2.999 D | 0.438 D | 2.382 C | 1.833 A | 2.384 A | 2.368 A | 33.797 A |
|  | (Co1) | 3.204 BC | 0.485 C | 2.630 B | 1.545 B | 2.020 B-D | 2.510 A | 28.228 B |
|  | (Co2) | 3.191 C | 0.483 C | 2.635 B | 1.510 B | 1.950 D | 2.588 A | 28.127 C |
|  | (Bs) | 3.229 B | 0.519 B | 2.754 A | 1.553 B | 1.995 CD | 2.364 A | 29.328 B |
|  | (Co1+ Bs) | 3.227 B | 0.502 B | 2.767 A | 1.558 B | 2.090 B | 2.546 A | 27.900 C |
|  | (Co2+ Bs) | 3.281 A | 0.543 A | 2.777 A | 1.505 B | 2.069 BC | 2.534 A | 25.759 D |
| The interaction between (SA × SP) | | | | | | | | |
| (S0) | (Cont.) | 3.556 b | 0.608 b | 3.265 a | 0.528 j | 0.698 ij | 6.313 a | 16.716 j |
|  | (Co1) | 3.605 ab | 0.635 ab | 3.338 a | 0.590 j | 0.808 h-j | 5.790 ab | 17.058 j |
|  | (Co2) | 3.563 b | 0.613 b | 3.281 a | 0.550 j | 0.656 j | 6.028 ab | 16.681 j |
|  | (Bs) | 3.631 a | 0.630 ab | 3.273 a | 0.676 j | 0.858 hi | 4.848 c | 17.203 j |
|  | (Co1+ Bs) | 3.573 ab | 0.615 b | 3.351 a | 0.586 j | 0.693 ij | 5.765 ab | 17.086 j |
|  | (Co2+ Bs) | 3.591 ab | 0.660 a | 3.331 a | 0.598 j | 0.911 h | 5.573 b | 16.306 j |
| (S1) | (Cont.) | 3.320 e | 0.426 f-h | 2.385 fg | 1.450 h | 2.075 e | 1.655 ef | 25.301 g |
|  | (Co1) | 3.355 de | 0.493 d | 2.563 d | 1.208 i | 1.488 g | 2.123 de | 22.633 h |
|  | (Co2) | 3.363 de | 0.470 de | 2.650 cd | 1.246 i | 1.553 g | 2.131 de | 22.413 h |
|  | (Bs) | 3.405 cd | 0.545 c | 2.811 b | 1.238 i | 1.780 f | 2.283 d | 21.968 h |
|  | (Co1+ Bs) | 3.378 de | 0.491 d | 2.743 bc | 1.300 hi | 1.915 ef | 2.118 de | 21.465 hi |
|  | (Co2+ Bs) | 3.453 c | 0.566 c | 2.800 b | 1.328 hi | 1.773 f | 2.108 de | 19.551 i |
| (S2) | (Cont.) | 2.761 ij | 0.376 j | 2.171 i | 2.296 bc | 3.075 b | 0.946 gh | 39.123 c |
|  | (Co1) | 3.078 g | 0.430 fg | 2.365 f-h | 2.001 fg | 2.605 d | 1.183 fg | 33.708 de |
|  | (Co2) | 3.060 g | 0.455 ef | 2.363 f-h | 1.958 g | 2.438 d | 1.211 fg | 33.745 de |
|  | (Bs) | 3.150 f | 0.488 de | 2.540 de | 2.066 efg | 2.495 d | 1.238 fg | 35.260 d |
|  | (Co1+ Bs) | 3.163 f | 0.475 de | 2.563 d | 2.098 d-g | 2.610 d | 1.226 fg | 32.528 e |
|  | (Co2+ Bs) | 3.195 f | 0.491 d | 2.585 d | 1.930 g | 2.475 d | 1.348 fg | 28.451 f |
| (S3) | (Cont.) | 2.358 k | 0.341 k | 1.710 j | 3.060 a | 3.688 a | 0.560 h | 54.050 a |
|  | (Co1) | 2.780 ij | 0.381 ij | 2.253 g-i | 2.381 b | 3.181 b | 0.946 gh | 39.513 c |
|  | (Co2) | 2.778 ij | 0.395 h-j | 2.245 hi | 2.288 bc | 3.153 b | 0.981 gh | 39.668 c |
|  | (Bs) | 2.730 j | 0.413 g-i | 2.393 f | 2.231 b-e | 2.846 c | 1.086 gh | 42.883 b |
|  | (Co1+ Bs) | 2.796 i | 0.428 f-h | 2.410 f | 2.248 b-d | 3.141 b | 1.076 gh | 40.523 c |
|  | (Co2+ Bs) | 2.885 h | 0.455 ef | 2.393 f | 2.163 c-f | 3.118 b | 1.108 f-h | 38.726 c |
| LSD_0.05_ (SA) | | 0.052 | 0.016 | 0.103 | 0.054 | 0.072 | 0.215 | 0.399 |
| LSD_0.05_ (SP) | | 0.033 | 0.017 | 0.067 | 0.085 | 0.088 | 0.278 | 0.967 |
| LSD_0.05_ (SA × SP) | | 0.066 | 0.034 | 0.134 | 0.171 | 0.176 | 0.557 | 1.935 |

LSD_0.05_ = least significant differences at 0.05 probability. Means with the same letters in the same column are not significantly different (P ≤ 0.05) according to Tukey's test. Nitrogen “N” (%), Phosphorus “P” (%), Potassium “K” (%), sodium “Na” (%), chlorine “Cl” (%), K:Na ratio, and Electrolyte leakage “EL” (%).

**Supplementary Tables S3.**

Influence of applied six salt stress protectants (SP); cobalt (Co1) at 15 ppm, (Co2) at 30 ppm, *Bacillus subtilis* (Bs), (Co1 + Bs), (Co2 + Bs), and distilled water as a control (Cont.) to irrigation water under four levels of NaCl irrigation water salinity (SA); tap water as a control (S0; 0.5, S1; 1.5, S2; 4 and S3; 6 dS m^−1^) and their interaction on Determine the activity of antioxidant enzymes, Ascorbic acid and Proline content in leaf of *Coriandrum sativum* L. cv. Balady. Values are in the form of means of both seasons 2022 and 2023.

| Treatments | | SOD  (U/mg protein) | CAT  (U /mg protein) | MDA  (nmol g f wt^-1^) | Asco  (mg 100 g f wt^-1^) | Pro  (µg 100 g d wt^-1^) |
| --- | --- | --- | --- | --- | --- | --- |
| The main plot (SA) | (S0) | 4.156 D | 0.306 D | 0.490 D | 32.908 A | 156.192 D |
|  | (S1) | 4.570 C | 0.357 C | 0.952 C | 29.358 B | 293.695 C |
|  | (S2) | 5.193 B | 0.446 B | 1.154 B | 28.038 C | 533.760 B |
|  | (S3) | 6.440 A | 0.573 A | 1.394 A | 25.472 D | 671.300 A |
| Main effect of subplot (SP) | (Cont.) | 5.278 A | 0.451 A | 1.102 A | 28.054 D | 441.777 A |
|  | (Co1) | 5.111 B | 0.432 B | 1.010 B | 28.612 CD | 415.445 B |
|  | (Co2) | 5.054 C | 0.421 C | 0.973 CD | 28.679 B-D | 437.456 A |
|  | (Bs) | 5.046 C | 0.414 CD | 0.986 C | 28.725 BC | 418.644 B |
|  | (Co1+ Bs) | 5.036 C | 0.407 DE | 0.965 D | 29.312 B | 388.356 C |
|  | (Co2+ Bs) | 5.011 C | 0.400 E | 0.949 E | 30.283 A | 380.743 C |
| The interaction between (SA × SP) | | | | | | |
| (S0) | (Cont.) | 4.151 kl | 0.291 m | 0.519 k | 32.333 bc | 164.698 k |
|  | (Co1) | 4.100 l | 0.308 lm | 0.488 l | 31.950 c | 149.259 k |
|  | (Co2) | 4.133 kl | 0.310 lm | 0.481 l | 32.266 bc | 159.804 k |
|  | (Bs) | 4.181 kl | 0.325 kl | 0.485 l | 32.800 bc | 155.641 k |
|  | (Co1+ Bs) | 4.150 kl | 0.306 lm | 0.486 l | 33.516 ab | 153.293 k |
|  | (Co2+ Bs) | 4.220 k | 0.300 m | 0.480 l | 34.583 a | 154.455 k |
| (S1) | (Cont.) | 4.753 h | 0.395 g | 1.185 d | 28.683 ef | 320.868 i |
|  | (Co1) | 4.628 i | 0.368 h | 0.970 h | 29.483 de | 283.905 j |
|  | (Co2) | 4.523 j | 0.358 hi | 0.880 j | 29.033 ef | 290.075 j |
|  | (Bs) | 4.536 ij | 0.346 ij | 0.923 i | 28.866 ef | 293.953 ij |
|  | (Co1+ Bs) | 4.513 j | 0.341 i-k | 0.891 j | 29.483 de | 275.291 j |
|  | (Co2+ Bs) | 4.465 j | 0.332 jk | 0.865 j | 30.600 d | 298.081 ij |
| (S2) | (Cont.) | 5.459 d | 0.508 d | 1.284 c | 26.666 gh | 593.768 de |
|  | (Co1) | 5.260 e | 0.465 e | 1.168 de | 27.950 fg | 548.962 fg |
|  | (Co2) | 5.172 ef | 0.448 e | 1.123 f | 28.316 ef | 573.049 ef |
|  | (Bs) | 5.121 fg | 0.423 f | 1.142 ef | 27.966 fg | 539.179 g |
|  | (Co1+ Bs) | 5.117 fg | 0.418 f | 1.116 fg | 28.200 ef | 485.354 h |
|  | (Co2+ Bs) | 5.028 g | 0.417 f | 1.090 g | 29.133 ef | 462.248 h |
| (S3) | (Cont.) | 6.750 a | 0.610 a | 1.421 a | 24.533 j | 687.773 b |
|  | (Co1) | 6.458 b | 0.588 b | 1.413 a | 25.066 ij | 679.656 b |
|  | (Co2) | 6.390 bc | 0.568 bc | 1.408 a | 25.100 ij | 726.897 a |
|  | (Bs) | 6.346 c | 0.563 c | 1.393 ab | 25.266 ij | 685.804 b |
|  | (Co1+ Bs) | 6.365 bc | 0.563 c | 1.368 b | 26.050 hi | 639.485 c |
|  | (Co2+ Bs) | 6.333 c | 0.550 c | 1.363 b | 26.816 gh | 608.187 d |
| LSD_0.05_ (SA) | | 0.027 | 0.010 | 0.011 | 0.458 | 14.019 |
| LSD_0.05_ (SP) | | 0.046 | 0.010 | 0.015 | 0.665 | 14.211 |
| LSD_0.05_ (SA × SP) | | 0.093 | 0.021 | 0.030 | 1.331 | 28.422 |

LSD_0.05_ = least significant differences at 0.05 probability. Means with the same letters in the same column are not significantly different (P ≤ 0.05) according to Tukey's test. superoxide dismutase activities “SOD” (U/mg protein), Catalase activities “CAT” (U /mg protein), Malondialdehyde “MDA” (nmol g f wt ^-1^), Ascorbic acid “Asco” (mg 100 g f wt ^-1^), and Proline “Pro” (µg 100 g d wt^-1^).

**Supplementary Tables S4.**

Influence of applied six salt stress protectants (SP); cobalt (Co1) at 15 ppm, (Co2) at 30 ppm, *Bacillus subtilis* (Bs), (Co1 + Bs), (Co2 + Bs), and distilled water as a control (Cont.) to irrigation water under four levels of NaCl irrigation water salinity (SA); tap water as a control (S0; 0.5, S1; 1.5, S2; 4 and S3; 6 dS m^−1^) and their interaction on important biological phytochemicals in seed oil of *Coriandrum sativum* L. cv. Balady. Values are in the form of means of both seasons 2022 and 2023.

| Treatments | | Linalool (%) | γ-terpinene (%) | α-pinene (%) | p-cymene (%) | Camphor (%) | Geranyl acetate (%) | Protein (%) |
| --- | --- | --- | --- | --- | --- | --- | --- | --- |
| The main plot (SA) | (S0) | 72.006 A | 3.978 C | 2.032 D | 2.539 D | 2.231 D | 1.818 D | 22.419 A |
|  | (S1) | 60.428 B | 4.176 B | 2.572 C | 3.009 C | 2.740 C | 2.143 C | 21.121 B |
|  | (S2) | 48.729 C | 4.21 B | 3.012 B | 3.133 B | 2.961 B | 2.283 B | 19.175 C |
|  | (S3) | 42.624 D | 4.813 A | 5.491 A | 3.613 A | 3.518 A | 2.707 A | 17.009 D |
| Main effect of subplot (SP) | (Cont.) | 53.010 D | 4.313 A | 3.442 A | 3.069 A | 2.818 B | 2.251 AB | 18.745 D |
|  | (Co1) | 54.942 C | 4.265 A | 3.228 BC | 3.050 A | 2.817 B | 2.232 AB | 20.029 BC |
|  | (Co2) | 55.519 C | 4.305 A | 3.281 B | 3.079 A | 2.830 B | 2.233 AB | 19.947 C |
|  | (Bs) | 56.652 B | 4.306 A | 3.244 BC | 3.083 A | 2.887 A | 2.264 A | 20.183 B |
|  | (Co1+ Bs) | 57.104 B | 4.301 A | 3.337 AB | 3.075 A | 2.902 A | 2.237 AB | 20.175 B |
|  | (Co2+ Bs) | 58.452 A | 4.275 A | 3.129 C | 3.083 A | 2.919 A | 2.210 B | 20.508 A |
| The interaction between (SA × SP) | | | | | | | | |
| (S0) | (Cont.) | 70.976 b | 3.981 fg | 2.238 hi | 2.540 j | 2.253 h | 1.826 gh | 22.230 b |
|  | (Co1) | 72.265 ab | 3.970 fg | 1.930 jk | 2.520 j | 2.205 h | 1.816 gh | 22.531 ab |
|  | (Co2) | 71.790 ab | 3.973 fg | 2.020 i-k | 2.551 j | 2.200 h | 1.810 gh | 22.273 b |
|  | (Bs) | 72.265 ab | 4.006 e-g | 2.030 i-k | 2.521 j | 2.230 h | 1.873 g | 22.698 a |
|  | (Co1+ Bs) | 72.050 ab | 3.988 fg | 2.130 ij | 2.535 j | 2.250 h | 1.831 gh | 22.333 ab |
|  | (Co2+ Bs) | 72.695 a | 3.951 g | 1.845 k | 2.566 j | 2.248 h | 1.751 h | 22.450 ab |
| (S1) | (Cont.) | 56.376 f | 4.183 cd | 2.730 ef | 3.055 e-g | 2.700 fg | 2.193 c-e | 20.750 e |
|  | (Co1) | 57.983 f | 4.210 cd | 2.525 fg | 3.045 fg | 2.754 fg | 2.165 e | 20.970 de |
|  | (Co2) | 59.711 e | 4.221 cd | 2.596 fg | 3.060 d-g | 2.801 ef | 2.146 ef | 21.023 de |
|  | (Bs) | 62.166 d | 4.206 cd | 2.561 fg | 3.005 gh | 2.760 fg | 2.168 de | 21.283 cd |
|  | (Co1+ Bs) | 62.458 cd | 4.135 c-e | 2.631 fg | 2.965 hi | 2.741 fg | 2.121 ef | 21.116 de |
|  | (Co2+ Bs) | 63.875 c | 4.101 d-f | 2.391 gh | 2.925 i | 2.683 g | 2.068 f | 21.583 c |
| (S2) | (Cont.) | 44.723 kl | 4.186 cd | 3.155 c | 3.123 c-e | 2.900 de | 2.251 b-d | 17.260 ij |
|  | (Co1) | 46.743 j | 4.190 cd | 2.940 c-e | 3.105 c-f | 2.932 d | 2.266 bc | 19.240 g |
|  | (Co2) | 48.386 ij | 4.243 c | 3.021 cd | 3.160 c | 2.946 d | 2.310 b | 19.126 g |
|  | (Bs) | 49.726 hi | 4.228 cd | 2.985 cd | 3.140 c | 2.987 d | 2.315 b | 19.688 f |
|  | (Co1+ Bs) | 50.566 gh | 4.205 cd | 3.070 cd | 3.138 c | 2.996 d | 2.286 b | 19.771 f |
|  | (Co2+ Bs) | 52.228 g | 4.206 cd | 2.905 de | 3.133 cd | 3.001 d | 2.271 bc | 19.968 f |
| (S3) | (Cont.) | 39.966 n | 4.901 a | 5.648 a | 3.560 b | 3.420 c | 2.733 a | 14.741 k |
|  | (Co1) | 42.780 m | 4.691 b | 5.518 ab | 3.533 b | 3.378 c | 2.680 a | 17.375 ij |
|  | (Co2) | 42.190 m | 4.783 ab | 5.488 ab | 3.545 b | 3.374 c | 2.668 a | 17.366 ij |
|  | (Bs) | 42.453 m | 4.783 ab | 5.400 b | 3.666 a | 3.573 b | 2.701 a | 17.063 j |
|  | (Co1+ Bs) | 43.343 lm | 4.878 a | 5.516 ab | 3.665 a | 3.621 b | 2.710 a | 17.480 i |
|  | (Co2+ Bs) | 45.011 k | 4.843 a | 5.375 b | 3.708 a | 3.742 a | 2.751 a | 18.031 h |
| LSD_0.05_ (SA) | | 0.559 | 0.040 | 0.071 | 0.056 | 0.064 | 0.041 | 0.328 |
| LSD_0.05_ (SP) | | 0.833 | 0.066 | 0.122 | 0.039 | 0.052 | 0.042 | 0.206 |
| LSD_0.05_ (SA × SP) | | 1.666 | 0.132 | 0.245 | 0.078 | 0.104 | 0.084 | 0.413 |

LSD_0.05_ = least significant differences at 0.05 probability. Means with the same letters in the same column are not significantly different (P ≤ 0.05) according to Tukey's test. Linalool “Linalool” (%), γ-terpinene “γ-terpinene” (%), α-pinene “α-pinene” (%), p-cymene “p-cymene” (%), Camphor “Camphor” (%), Geranyl acetate “Geranyl acetate” (%), and Protein (%) “protein” (%).

**Supplementary Tables S5.**

Influence of applied six salt stress protectants (SP); cobalt (Co1) at 15 ppm, (Co2) at 30 ppm, *Bacillus subtilis* (Bs), (Co1 + Bs), (Co2 + Bs), and distilled water as a control (Cont.) to irrigation water under four levels of NaCl irrigation water salinity (SA); tap water as a control (S0; 0.5, S1; 1.5, S2; 4 and S3; 6 dS m^−1^) and their interaction on yield parameters of seed and oil of *Coriandrum sativum* L. cv. Balady. Values are in the form of means of both seasons 2022 and 2023.

| Treatments | | SYP (g) | LO (%) | EOYS (%) |
| --- | --- | --- | --- | --- |
|  |  |  |  |  |
| The main plot (SA) | (S0) | 3.427 A | 0.123 C | 0.594 A |
|  | (S1) | 3.236 B | 0.135 B | 0.520 B |
|  | (S2) | 2.796 C | 0.140 AB | 0.483 C |
|  | (S3) | 1.968 D | 0.144 A | 0.384 D |
| Main effect of subplot (SP) | (Cont.) | 2.683 E | 0.141 A | 0.447 D |
|  | (Co1) | 2.777 D | 0.134 B | 0.477 C |
|  | (Co2) | 2.798 D | 0.137 AB | 0.499 B |
|  | (Bs) | 2.895 C | 0.132 B | 0.496 BC |
|  | (Co1+ Bs) | 2.968 B | 0.137 AB | 0.523 A |
|  | (Co2+ Bs) | 3.02 A | 0.132 B | 0.531 A |
| The interaction between (SA × SP) | | | | |
| (S0) | (Cont.) | 3.345 c | 0.125 f-i | 0.543 de |
|  | (Co1) | 3.418 b | 0.125 f-i | 0.580 cd |
|  | (Co2) | 3.411 b | 0.123 hi | 0.591 bc |
|  | (Bs) | 3.430 b | 0.124 g-i | 0.588 bc |
|  | (Co1+ Bs) | 3.435 b | 0.125 f-i | 0.626 ab |
|  | (Co2+ Bs) | 3.525 a | 0.116 i | 0.636 a |
| (S1) | (Cont.) | 3.145 g | 0.141 b-e | 0.488 fg |
|  | (Co1) | 3.211 f | 0.138 b-e | 0.503 f |
|  | (Co2) | 3.215 f | 0.137 b-f | 0.518 ef |
|  | (Bs) | 3.246 ef | 0.131 d-h | 0.508 ef |
|  | (Co1+ Bs) | 3.281 de | 0.135 c-h | 0.543 de |
|  | (Co2+ Bs) | 3.316 cd | 0.130 e-h | 0.563 cd |
| (S2) | (Cont.) | 2.528 l | 0.143 b-d | 0.421 h-j |
|  | (Co1) | 2.703 k | 0.133 c-h | 0.460 gh |
|  | (Co2) | 2.773 j | 0.141 b-e | 0.505 ef |
|  | (Bs) | 2.873 i | 0.141 b-e | 0.503 f |
|  | (Co1+ Bs) | 2.933 h | 0.145 a-c | 0.515 ef |
|  | (Co2+ Bs) | 2.968 h | 0.136 b-g | 0.498 fg |
| (S3) | (Cont.) | 1.716 p | 0.156 a | 0.336 m |
|  | (Co1) | 1.776 o | 0.141 b-e | 0.365 lm |
|  | (Co2) | 1.795 o | 0.148 ab | 0.381 kl |
|  | (Bs) | 2.030 n | 0.131 d-h | 0.385 j-l |
|  | (Co1+ Bs) | 2.225 m | 0.145 a-c | 0.410 i-k |
|  | (Co2+ Bs) | 2.270 m | 0.145 a-c | 0.428 hi |
| LSD_0.05_ (SA) | | 0.026 | 0.007 | 0.008 |
| LSD_0.05_ (SP) | | 0.029 | 0.006 | 0.019 |
| LSD_0.05_ (SA × SP) | | 0.058 | 0.013 | 0.039 |

LSD_0.05_ = least significant differences at 0.05 probability. Means with the same letters in the same column are not significantly different (P ≤ 0.05) according to Tukey's test. Seed yield per plant “SYP” (g), Leaf oil “LO” (%), and Essential oil yield “EOYS” (%)
