# Supplementary material for: Promoting salt tolerance, growth, and phytochemical responses in coriander (Coriandrum sativum L. cv. Balady) via eco-friendly Bacillus subtilis and cobalt
Source: BMC Plant Biol. 2024 Sep 10;24:848. doi: 10.1186/s12870-024-05517-3 (PMC11384715; doi:10.1186/s12870-024-05517-3)
Supplement: Supplementary file 2 — Additional file 2. [file 12870_2024_5517_MOESM2_ESM.docx]

6 X 4 = 24 Treatments **Two seasons (separate)**

**S1** Growth parameters and characteristics

1. Number of leaves “NL”
2. Leaf length “LL” (cm)
3. Shoot fresh weight “SFW” (g)
4. Root fresh weight “RFW” (g)
5. Root dry weight “RDW” (g)
6. Plant fresh weight “PFW” (g)
7. Plant dry weight “PDW” (g)
8. Plant height “Ph” (cm)
9. Chlorophyll content (SPAD Unit)

**Chemical measurements and analyses**

**S2** Elements content dry leaves

1. Nitrogen “N” (%)
2. Phosphorus “P” (%)
3. Potassium “K” (%)
4. Sodium “Na” (%)
5. Chlorine “Cl” (%)
6. K:Na ratio
7. Electrolyte leakage “EL” (%)

**S3** Determine the activity of antioxidant enzymes, Ascorbic acid, and Proline content in leaf 

1. Specific activity of superoxide dismutase “SOD” (U/mg protein)
2. Catalase activities “CAT” (U /mg protein)
3. Malondialdehyde “MDA” (nmol/g F.wt.), = MDA (nmol g f wt^-1^)
4. Ascorbic acid “Asco” (mg/100 g f. wt.)
5. Proline “Pro” (µg /100 g D. wt.)

**S4** Percentage of important biological phytochemicals in coriander seed oil

1. Linalool “Linalool” (%)
2. γ-terpinene “γ-terpinene” (%)
3. α-pinene “α-pinene” (%)
4. p-cymene “p-cymene” (%)
5. Camphor “Camphor” (%)
6. Geranyl acetate “Geranyl acetate” (%)
7. Protein %

**S5** Yield parameters of seed and oil

1. Seed yield per plant “SYP” (g)
2. Leaf oil “LO” (%)
3. Essential oil yield “EOYS” (%)

**Supplementary Tables S1.**

Influence of applied six salt stress protectants (SP); cobalt (Co1) at 15 ppm, (Co2) at 30 ppm, *Bacillus subtilis* (Bs), (Co1 + Bs), (Co2 + Bs), and distilled water as a control (Cont.) to irrigation water under four levels of NaCl irrigation water salinity (SA); tap water as a control (S0; 0.5, S1; 1.5, S2; 4 and S3; 6 dS m^−1^) and their interaction on growth parameters and characteristics of *Coriandrum sativum* L. cv. Balady. Values are in the form of means of two seasons 2022 and 2023 separate.

| Treatments | | NL | | LL (cm) | | SFW (g) | | RFW (g) | |
| --- | --- | --- | --- | --- | --- | --- | --- | --- | --- |
|  |  | 2022 | 2023 | 2022 | 2023 | 2022 | 2023 | 2022 | 2023 |
| The main plot (SA) | (S0) | 15.39 A | 16.67 A | 22.21 A | 21.56 A | 14.02 A | 13.73 A | 5.29 A | 5.67 A |
|  | (S1) | 14.17 B | 14.33 B | 19.13 B | 19.64 B | 11.28 B | 11.35 B | 4.64 B | 5.02 B |
|  | (S2) | 13.94 B | 13.78 B | 17.43 C | 18.39 C | 7.75 C | 8.56 C | 3.73 C | 4.25 C |
|  | (S3) | 9.77 C | 10.17 C | 12.04 D | 16.02 D | 5.85 D | 6.83 D | 3.27 D | 3.76 D |
| Main effect of subplot (SP) | (Cont.) | 9.83 E | 11.00 D | 13.49 C | 16.89 C | 7.55 D | 8.29 E | 3.70 D | 4.25 D |
|  | (Co1) | 12.00 D | 12.92 C | 18.16 B | 18.71 B | 9.48 C | 9.54 D | 4.12 C | 4.65 C |
|  | (Co2) | 13.42 C | 13.33 BC | 19.17 A | 19.31 AB | 10.29 B | 10.41 C | 4.22 BC | 4.62 C |
|  | (Bs) | 14.42 B | 14.17 B | 18.11 B | 19.14 AB | 9.72 C | 10.61 BC | 4.30 B | 4.62 C |
|  | (Co1+ Bs) | 15.75 A | 15.33 A | 18.58 AB | 19.69 A | 10.13 BC | 10.79 AB | 4.30 B | 4.89 B |
|  | (Co2+ Bs) | 14.50 B | 15.33 A | 18.81 AB | 19.68 A | 11.19 A | 11.06 A | 4.67 A | 5.02 A |
| The interaction between (SA × SP) | | | | | | | | | |
| (S0) | (Cont.) | 14.00 e-g | 15.67 b-e | 20.67 b-e | 20.90 a-f | 12.22 de | 12.56 cd | 5.03 b-d | 5.38 cd |
|  | (Co1) | 14.00 e-g | 15.67 b-e | 22.43 ab | 21.97 ab | 13.73 bc | 12.97 c | 5.19 bc | 5.56 bc |
|  | (Co2) | 15.00 c-f | 16.33 a-d | 24.17 a | 21.57 a-c | 13.85 bc | 13.73 b | 5.14 bc | 5.61 bc |
|  | (Bs) | 16.00 a-e | 17.33 ab | 21.50 b-d | 21.37 a-c | 14.40 ab | 14.14 ab | 5.22 b | 5.61 bc |
|  | (Co1+ Bs) | 16.33 a-d | 17.33 ab | 22.07 a-c | 21.17 a-d | 14.51 ab | 14.58 a | 5.29 b | 5.79 b |
|  | (Co2+ Bs) | 17.00 a-c | 17.67 a | 22.43 ab | 22.40 a | 15.44 a | 14.43 a | 5.89 a | 6.09 a |
| (S1) | (Cont.) | 11.67 h-j | 12.00 h-j | 14.37 h | 17.30 h-j | 9.91 gh | 9.12 g | 3.72 gh | 4.75 f |
|  | (Co1) | 12.67 g-i | 14.00 e-g | 19.63 d-g | 18.87 f-h | 10.90 fg | 10.46 f | 4.63 e | 5.16 de |
|  | (Co2) | 14.33 d-g | 14.33 e-g | 20.47 b-f | 19.90 c-g | 12.21 de | 11.88 e | 4.69 de | 5.03 e |
|  | (Bs) | 15.33 b-f | 14.67 d-f | 19.50 d-g | 19.93 b-g | 10.79 fg | 11.75 e | 4.85 c-e | 5.04 e |
|  | (Co1+ Bs) | 17.33 ab | 15.67 b-e | 20.70 b-e | 21.03 a-e | 11.19 ef | 12.09 de | 4.95 b-e | 5.05 e |
|  | (Co2+ Bs) | 13.67 f-h | 15.33 c-e | 20.10 c-f | 20.80 a-f | 12.67 cd | 12.80 c | 5.00 b-d | 5.10 e |
| (S2) | (Cont.) | 8.67 k | 10.00 kl | 11.57 i | 15.73 j | 5.02 k | 6.73 j | 3.20 jk | 3.62 h |
|  | (Co1) | 11.33 ij | 12.67 g-i | 17.83 g | 18.20 g-i | 7.07 ij | 7.87 h | 3.37 ij | 4.15 g |
|  | (Co2) | 14.00 e-g | 13.33 f-h | 18.90 e-g | 19.03 e-h | 8.91 h | 8.97 g | 3.70 h | 4.09 g |
|  | (Bs) | 15.00 c-f | 13.33 f-h | 18.30 fg | 18.90 f-h | 7.51 i | 9.27 g | 3.93 f-h | 4.08 g |
|  | (Co1+ Bs) | 18.00 a | 16.33 a-d | 18.90 e-g | 19.30 d-h | 8.75 h | 9.23 g | 4.05 fg | 4.74 f |
|  | (Co2+ Bs) | 16.67 a-c | 17.00 a-c | 19.10 e-g | 19.20 d-h | 9.23 h | 9.27 g | 4.10 f | 4.79 f |
| (S3) | (Cont.) | 5.00 l | 6.33 m | 7.37 j | 13.63 k | 3.04 l | 4.77 k | 2.84 l | 3.24 i |
|  | (Co1) | 10.00 jk | 9.33 l | 12.73 hi | 15.80 j | 6.20 jk | 6.85 j | 3.27 jk | 3.74 h |
|  | (Co2) | 10.33 jk | 10.67 j-l | 13.13 hi | 16.73 ij | 6.20 jk | 7.03 j | 3.36 ij | 3.75 h |
|  | (Bs) | 11.33 ij | 11.33 i-k | 12.67 hi | 16.37 ij | 6.05 jk | 7.27 ij | 3.19 jk | 3.75 h |
|  | (Co1+ Bs) | 11.33 ij | 12.00 h- | 12.20 hi | 17.27 h-j | 5.55 k | 7.31 h-j | 2.93 kl | 4.00 g |
|  | (Co2+ Bs) | 10.67 i-k | 11.33 i-k | 13.60 hi | 16.33 ij | 7.42 i | 7.73 hi | 3.68 hi | 4.08 g |
| LSD_0.05_ (SA) | | 0.635 | 0.774 | 0.794 | 0.737 | 0.513 | 0.199 | 0.087 | 0.077 |
| LSD_0.05_ (SP) | | 0.956 | 0.774 | 0.984 | 0.886 | 0.564 | 0.274 | 0.158 | 0.1104 |
| LSD_0.05_ (SA × SP) | | 1.91 | 1.548 | 1.967 | 1.77 | 1.127 | 0.55 | 0.316 | 0.220 |

LSD_0.05_ = least significant differences at 0.05 probability. Means with the same letters in the same column are not significantly different (P ≤ 0.05) according to Tukey's test. Number of leaves “NL”, Leaf length “LL” (cm), shoot fresh weight “SFW” (g), and root fresh weight “RFW” (g).

**Follow - Supplementary Tables S1.**

Influence of applied six salt stress protectants (SP); cobalt (Co1) at 15 ppm, (Co2) at 30 ppm, *Bacillus subtilis* (Bs), (Co1 + Bs), (Co2 + Bs), and distilled water as a control (Cont.) to irrigation water under four levels of NaCl irrigation water salinity (SA); tap water as a control (S0; 0.5, S1; 1.5, S2; 4 and S3; 6 dS m^−1^) and their interaction on growth parameters and characteristics of *Coriandrum sativum* L. cv. Balady. Values are in the form of means of two seasons 2022 and 2023 separate.

| Treatments | | RDW (g) | | PFW (g) | | PDW (g) | | Ph (cm) | | Chlorophyll (SPAD Unit) | |
| --- | --- | --- | --- | --- | --- | --- | --- | --- | --- | --- | --- |
|  |  | 2022 | 2023 | 2022 | 2023 | 2022 | 2023 | 2022 | 2023 | 2022 | 2023 |
| The main plot (SA) | (S0) | 0.90 A | 0.79 A | 42.07 A | 41.20 A | 5.47A | 5.36 A | 86.94 A | 86.56 A | 44.22 A | 43.22 A |
|  | (S1) | 0.84 B | 0.75 B | 33.84 B | 34.05 B | 4.74 B | 4.70 B | 80.22 B | 78.93 B | 42.39 B | 40.72 B |
|  | (S2) | 0.71 C | 0.72 C | 23.25 C | 25.63 C | 3.37 C | 3.85 C | 71.20 C | 70.22 C | 40.28 C | 40.56 B |
|  | (S3) | 0.65 D | 0.67 D | 17.55 D | 20.25 D | 2.63 D | 3.48 D | 64.23 D | 65.58 D | 39.39 D | 38.50 C |
| Main effect of subplot (SP) | (Cont.) | 0.68 D | 0.67 D | 22.64 D | 24.88 D | 3.12 D | 3.53 E | 72.10 D | 71.03 E | 40.08 C | 38.75 C |
|  | (Co1) | 0.75 C | 0.73 C | 28.44 C | 28.61 C | 3.95 C | 4.11 D | 76.11 B | 75.16 D | 41.58 B | 40.67 B |
|  | (Co2) | 0.77 BC | 0.73 C | 30.87 B | 31.02 B | 4.30 B | 4.44 C | 77.73 A | 76.30 BC | 42.00 AB | 41.17 AB |
|  | (Bs) | 0.79 B | 0.73 C | 29.15 C | 31.64 B | 4.03 C | 4.53 BC | 75.00 C | 75.84 CD | 41.58 B | 41.00 AB |
|  | (Co1+ Bs) | 0.80 B | 0.77 B | 30.38 BC | 32.37 AB | 4.22 BC | 4.64 B | 74.20 C | 76.80 AB | 41.50 B | 41.25 AB |
|  | (Co2+ Bs) | 0.85 A | 0.79 A | 33.57 A | 33.18 A | 4.67 A | 4.76 A | 77.91 A | 77.17 A | 42.33 B | 41.67 A |
| The interaction between (SA × SP) | | | | | | | | | | | |
| (S0) | (Cont.) | 0.86 bc | 0.75 d-f | 36.65 de | 37.67 cd | 4.76 de | 4.90 c | 83.23 c | 84.00 d | 43.33 b-d | 43.00 a-d |
|  | (Co1) | 0.88 bc | 0.78 b-d | 41.20 bc | 38.90 c | 5.36 bc | 5.06 c | 86.28 b | 86.67 bc | 44.67 ab | 44.33 a |
|  | (Co2) | 0.87 bc | 0.79 b-d | 41.54 bc | 41.20 b | 5.40 bc | 5.36 b | 88.19 ab | 86.60 bc | 44.67 ab | 42.00 c-f |
|  | (Bs) | 0.89 b | 0.79 b-d | 43.20 ab | 42.41 ab | 5.62 ab | 5.51 ab | 87.16 b | 85.70 c | 43.67 a-d | 42.67 a-e |
|  | (Co1+ Bs) | 0.90 b | 0.81 b | 43.53 ab | 43.73 a | 5.66 ab | 5.68 a | 87.31 ab | 87.80 ab | 44.00 a-c | 43.33 a-c |
|  | (Co2+ Bs) | 1.00 a | 0.85 a | 46.31 a | 43.30 a | 6.02 a | 5.63 a | 89.51 a | 88.60 a | 45.00 a | 44.00 ab |
| (S1) | (Cont.) | 0.67 gh | 0.71 gh | 29.74 gh | 27.36 g | 4.16 fg | 3.78 f-h | 77.33 e | 74.40 g | 41.33 f-i | 39.33 h-j |
|  | (Co1) | 0.83 cd | 0.77 cd | 32.71 ef | 31.39 f | 4.58 ef | 4.33 d | 81.54 cd | 79.47 ef | 41.67 e-h | 39.00 ij |
|  | (Co2) | 0.84 bc | 0.75 d-f | 36.64 de | 35.65 e | 5.13 cd | 4.92 c | 82.20 c | 78.60 f | 42.33 d-g | 41.33 d-g |
|  | (Bs) | 0.87 bc | 0.76 de | 32.36 fg | 35.26 e | 4.53 ef | 4.87 c | 79.22 de | 79.83 ef | 43.00 c-e | 42.00 c-f |
|  | (Co1+ Bs) | 0.89 b | 0.76 de | 33.56 ef | 36.26 de | 4.70 de | 5.00 c | 79.59 de | 81.10 e | 42.67 c-f | 41.00 e-h |
|  | (Co2+ Bs) | 0.90 b | 0.76 de | 38.00 cd | 38.39 c | 5.32 bc | 5.30 b | 81.45 cd | 80.17 ef | 43.33 b-d | 41.67 c-f |
| (S2) | (Cont.) | 0.61 i-k | 0.62 j | 15.06 k | 20.20 k | 2.18 m | 3.03 j | 68.22 hi | 66.30 j | 39.00 kl | 37.00 kl |
|  | (Co1) | 0.64 h-j | 0.70 g-i | 21.22 ij | 23.60 h | 3.08 i-k | 3.54 i | 71.76 fg | 68.43 i | 40.00 i-l | 40.33 f-i |
|  | (Co2) | 0.70 fg | 0.69 hi | 26.74 h | 26.60 g | 3.88 g | 4.00 ef | 73.60 f | 72.20 h | 40.67 h-j | 42.33 b-e |
|  | (Bs) | 0.75 ef | 0.69 hi | 22.52 i | 27.82 g | 3.27 ij | 4.17 de | 70.63 g | 71.50 h | 40.33 h-k | 40.33 f-i |
|  | (Co1+ Bs) | 0.77 e | 0.80 bc | 26.26 h | 27.68 g | 3.81 gh | 4.15 de | 69.52 gh | 70.73 h | 40.67 h-j | 42.00 c-f |
|  | (Co2+ Bs) | 0.78 de | 0.81 b | 27.70 h | 27.81 g | 4.01 g | 4.17 de | 73.46 f | 72.13 h | 41.00 g-i | 41.33 d-g |
| (S3) | (Cont.) | 0.57 k | 0.58 j | 9.11 l | 14.30 l | 1.37 n | 2.43 k | 59.62 k | 59.43 k | 36.67 m | 35.67 l |
|  | (Co1) | 0.65 g-i | 0.67 i | 18.61 jk | 20.55 jk | 2.79 j-l | 3.49 i | 64.87 j | 66.07 j | 40.00 i-l | 39.00 ij |
|  | (Co2) | 0.67 gh | 0.68 i | 18.54 jk | 21.08 jk | 2.78 kl | 3.58 hi | 66.95 ij | 67.83 ij | 40.33 h-k | 39.00 ij |
|  | (Bs) | 0.64 h-j | 0.68 i | 18.15 jk | 21.80 i-k | 2.72 kl | 3.70 hi | 61.83 k | 66.33 j | 39.33 j-l | 39.00 ij |
|  | (Co1+ Bs) | 0.60 i-k | 0.72 f-h | 16.65 k | 21.92 ij | 2.50 lm | 3.73 g-i | 60.30 k | 67.57 ij | 38.67 l | 38.67 i-k |
|  | (Co2+ Bs) | 0.74 ef | 0.73 e-g | 22.26 i | 23.20 hi | 3.34 hi | 3.94 e-g | 67.22 i | 67.77 ij | 40.00 i-l | 39.67 g-i |
| LSD_0.05_ (SA) | | 0.013 | 0.0106 | 1.540 | 0.573 | 0.209 | 0.080 | 0.464 | 0.65 | 0.523 | 0.787 |
| LSD_0.05_ (SP) | | 0.028 | 0.018 | 1.691 | 0.872 | 0.240 | 0.117 | 1.083 | 0.793 | 0.686 | 0.90 |
| LSD_0.05_ (SA × SP) | | 0.057 | 0.036 | 3.383 | 2.04 | 0.481 | 0.234 | 2.165 | 1.587 | 1.372 | 1.799 |

LSD_0.05_ = least significant differences at 0.05 probability. Means with the same letters in the same column are not significantly different (P ≤ 0.05) according to Tukey's test. Root dry weight “RDW” (g), plant fresh weight “PFW” (g), plant dry weight “PDW” (g), plant height “Ph” (cm), and Chlorophyll content (SPAD Unite).

**Supplementary Tables S2.**

Influence of applied six salt stress protectants (SP); cobalt (Co1) at 15 ppm, (Co2) at 30 ppm, *Bacillus subtilis* (Bs), (Co1 + Bs), (Co2 + Bs), and distilled water as a control (Cont.) to irrigation water under four levels of NaCl irrigation water salinity (SA); tap water as a control (S0; 0.5, S1; 1.5, S2; 4 and S3; 6 dS m^−1^) and their interaction on elements content of dry leaves in *Coriandrum sativum* L. cv. Balady. Values are in the form of means of two seasons 2022 and 2023 separate.

| Treatments | | N (%) | | P (%) | | K (%) | | Na (%) | | Cl (%) | |
| --- | --- | --- | --- | --- | --- | --- | --- | --- | --- | --- | --- |
|  |  | 2022 | 2023 | 2022 | 2023 | 2022 | 2023 | 2022 | 2023 | 2022 | 2023 |
| The main plot (SA) | (S0) | 3.624 A | 3.550 A | 0.632 A | 0.622 A | 3.326 A | 3.288 A | 0.581 D | 0.596 D | 0.781 D | 0.761 D |
|  | (S1) | 3.363 B | 3.395 B | 0.492 B | 0.506 B | 2.637 B | 2.681 B | 1.278 C | 1.313 C | 1.750 C | 1.778 C |
|  | (S2) | 3.049 C | 3.087 C | 0.444 C | 0.461 C | 2.318 C | 2.544 C | 2.122 B | 1.996 B | 2.724 B | 2.508 B |
|  | (S3) | 2.628 D | 2.815 D | 0.409 D | 0.396 D | 2.148 D | 2.320 D | 2.447 A | 2.344 A | 3.309 A | 3.067 A |
| Main effect of subplot (SP) | (Cont.) | 2.95 C | 3.05 D | 0.45 D | 0.43 D | 2.38 C | 2.39 C | 1.85 A | 1.82 A | 2.50 A | 2.27 A |
|  | (Co1) | 3.18 B | 3.23 BC | 0.49 C | 0.48 BC | 2.60 B | 2.66 B | 1.55 B | 1.54 B | 2.07 B-D | 1.97 BC |
|  | (Co2) | 3.19 B | 3.19 C | 0.50 BC | 0.47 C | 2.63 AB | 2.64 B | 1.54 B | 1.49 B | 1.98 D | 1.92 C |
|  | (Bs) | 3.21 B | 3.25 B | 0.50 BC | 0.54 A | 2.70 A | 2.81 A | 1.59 B | 1.52 B | 2.03 CD | 1.96 BC |
|  | (Co1+ Bs) | 3.21 B | 3.25 B | 0.51 B | 0.50 B | 2.67 AB | 2.87 A | 1.58 B | 1.54 B | 2.15 B | 2.03 B |
|  | (Co2+ Bs) | 3.26 A | 3.30 A | 0.52 A | 0.56 A | 2.67 AB | 2.89 A | 1.54 B | 1.47 B | 2.11 BC | 2.03 B |
| The interaction between (SA × SP) | | | | | | | | | | | |
| (S0) | (Cont.) | 3.627 ab | 3.487 b-d | 0.630 a-c | 0.587 c | 3.240 a | 3.290 a | 0.553 i | 0.503 j | 0.733 k-m | 0.663 j |
|  | (Co1) | 3.637 ab | 3.573 ab | 0.640 ab | 0.630 a-c | 3.383 a | 3.293 a | 0.523 i | 0.657 j | 0.790 k-m | 0.827 ij |
|  | (Co2) | 3.597 b | 3.530 a-c | 0.623 bc | 0.603 bc | 3.367 a | 3.197 a | 0.550 i | 0.550 j | 0.657 m | 0.657 j |
|  | (Bs) | 3.673 a | 3.590 a | 0.613 c | 0.647 ab | 3.243 a | 3.303 a | 0.670 i | 0.683 j | 0.880 kl | 0.837 ij |
|  | (Co1+ Bs) | 3.607 ab | 3.540 a-c | 0.633 a-c | 0.597 bc | 3.387 a | 3.317 a | 0.593 i | 0.580 j | 0.700 lm | 0.687 j |
|  | (Co2+ Bs) | 3.603 ab | 3.580 ab | 0.653 a | 0.667 a | 3.333 a | 3.330 a | 0.597 i | 0.600 j | 0.927 k | 0.897 i |
| (S1) | (Cont.) | 3.310 e | 3.330 fg | 0.440 h-j | 0.413 h-k | 2.403 ef | 2.367g-i | 1.440 g | 1.460 h | 2.060 g | 2.090 e |
|  | (Co1) | 3.317 e | 3.393 d-f | 0.497 d-f | 0.490 d-f | 2.573 cd | 2.553 ef | 1.220 h | 1.197 i | 1.530 j | 1.447 h |
|  | (Co2) | 3.353 de | 3.373 ef | 0.500 d-f | 0.440 f-i | 2.660 c | 2.640 c-e | 1.217 h | 1.277 hi | 1.517 j | 1.590 gh |
|  | (Bs) | 3.407 cd | 3.403 d-f | 0.490 ef | 0.600 bc | 2.840 b | 2.783 bc | 1.180 h | 1.297 hi | 1.697 ij | 1.863 f |
|  | (Co1+ Bs) | 3.357 de | 3.400 d-f | 0.507 de | 0.477 d-g | 2.623 c | 2.863 b | 1.267 h | 1.333 hi | 1.903 gh | 1.927 ef |
|  | (Co2+ Bs) | 3.437 c | 3.470 c-e | 0.517 d | 0.617 a-c | 2.723 bc | 2.877 b | 1.343 gh | 1.313 hi | 1.793 hi | 1.753 fg |
| (S2) | (Cont.) | 2.790 h | 2.733 lm | 0.373 m | 0.380 j-l | 2.140 i | 2.203 j | 2.350 bc | 2.243 bc | 3.147 bc | 3.003 b |
|  | (Co1) | 3.067 f | 3.090 ij | 0.437 i-k | 0.423 g-j | 2.250 f-i | 2.480 e-g | 2.043 d-f | 1.960 d-g | 2.677 ef | 2.533 cd |
|  | (Co2) | 3.087 f | 3.033 j | 0.457g-i | 0.453 e-i | 2.290 f-i | 2.437 f-h | 2.017 ef | 1.900 fg | 2.493 f | 2.383 d |
|  | (Bs) | 3.113 f | 3.187 hi | 0.463 gh | 0.513 d | 2.463 de | 2.617 de | 2.187 c-e | 1.947 e-g | 2.587 f | 2.403 d |
|  | (Co1+ Bs) | 3.116 f | 3.210 h | 0.457 g-i | 0.493 d-f | 2.393 e-g | 2.733 b-d | 2.130 d-f | 2.067 c-f | 2.853 de | 2.367 d |
|  | (Co2+ Bs) | 3.120 f | 3.270 gh | 0.480 fg | 0.503 de | 2.373 e-h | 2.797 bc | 2.003 f | 1.857 g | 2.590 f | 2.360 d |
| (S3) | (Cont.) | 2.063 k | 2.653 m | 0.337 n | 0.347 l | 1.723 j | 1.697 k | 3.060 a | 3.060 a | 4.057 a | 3.320 a |
|  | (Co1) | 2.697 ij | 2.863 k | 0.403 l | 0.360 kl | 2.200 i | 2.307 h-j | 2.403 b | 2.360 b | 3.293 b | 3.070 b |
|  | (Co2) | 2.727 hi | 2.830 kl | 0.413 kl | 0.377 j-l | 2.220 hi | 2.270 ij | 2.357 bc | 2.220 bc | 3.247 b | 3.060 b |
|  | (Bs) | 2.647 j | 2.813 kl | 0.423 j-l | 0.403 i-k | 2.240 g-i | 2.547 ef | 2.327 bc | 2.137 c-e | 2.967 cd | 2.727 c |
|  | (Co1+ Bs) | 2.757 hi | 2.837 k | 0.433 i-k | 0.423 g-j | 2.260 f-i | 2.560 ef | 2.337 bc | 2.160 b-d | 3.153 bc | 3.130 ab |
|  | (Co2+ Bs) | 2.877 g | 2.893 k | 0.447 h-j | 0.463 d-h | 2.247 f-i | 2.540 ef | 2.200 cd | 2.127 c-e | 3.140 bc | 3.097 b |
| LSD_0.05_ (SA) | | 0.039 | 0.076 | 0.014 | 0.022 | 0.113 | 0.112 | 0.056 | 0.062 | 0.081 | 0.077 |
| LSD_0.05_ (SP) | | 0.038 | 0.048 | 0.0128 | 0.027 | 0.079 | 0.080 | 0.085 | 0.101 | 0.100 | 0.100 |
| LSD_0.05_ (SA × SP) | | 0.076 | 0.097 | 0.025 | 0.055 | 0.159 | 0.160 | 0.170 | 0.202 | 0.200 | 0.201 |

LSD_0.05_ = least significant differences at 0.05 probability. Means with the same letters in the same column are not significantly different (P ≤ 0.05) according to Tukey's test. Nitrogen “N” (%), Phosphorus “P” (%), Potassium “K” (%), sodium “Na” (%), and chlorine “Cl” (%).

**Follow - Supplementary Tables S2.**

Influence of applied six salt stress protectants (SP); cobalt (Co1) at 15 ppm, (Co2) at 30 ppm, *Bacillus subtilis* (Bs), (Co1 + Bs), (Co2 + Bs), and distilled water as a control (Cont.) to irrigation water under four levels of NaCl irrigation water salinity (SA); tap water as a control (S0; 0.5, S1; 1.5, S2; 4 and S3; 6 dS m^−1^) and their interaction on K:Na ratio and electrolyte leakage of dry leaves in *Coriandrum sativum* L. cv. Balady. Values are in the form of means of two seasons 2022 and 2023 separate.

| Treatments | | K:Na ratio | | EL (%) | |
| --- | --- | --- | --- | --- | --- |
|  |  | 2022 | 2023 | 2022 | 2023 |
| The main plot (SA) | (S0) | 5.796 A | 5.644 A | 16.308 D | 17.376 D |
|  | (S1) | 2.083 B | 2.057 B | 22.367 C | 22.078 C |
|  | (S2) | 1.100 C | 1.285 C | 35.426 B | 32.180 B |
|  | (S3) | 0.896 D | 1.024 C | 42.043 A | 43.078 A |
| Main effect of subplot (SP) | (Cont.) | 2.26 C | 2.47 A | 33.60 A | 33.99 A |
|  | (Co1) | 2.66 A | 2.36 A | 28.19 C | 28.26 B |
|  | (Co2) | 2.60 AB | 2.57 A | 27.90 C | 28.35 B |
|  | (Bs) | 2.34 BC | 2.39 A | 29.93 B | 28.73 B |
|  | (Co1+ Bs) | 2.49 A-C | 2.61 A | 28.04 C | 27.77 B |
|  | (Co2+ Bs) | 2.46 A-C | 2.61 A | 26.56 D | 24.96 C |
| The interaction between (SA × SP) | | | | | |
| (S0) | (Cont.) | 5.903 bc | 6.723 a | 16.140 l | 17.293 i |
|  | (Co1) | 6.523 a | 5.057 cd | 15.697 l | 18.420 i |
|  | (Co2) | 6.140 ab | 5.917 b | 15.717 l | 17.647 i |
|  | (Bs) | 4.853 d | 4.843 d | 16.733 l | 17.673 i |
|  | (Co1+ Bs) | 5.760 bc | 5.770 b | 17.290 l | 16.883 i |
|  | (Co2+ Bs) | 5.593 c | 5.553 bc | 16.273 l | 16.340 i |
| (S1) | (Cont.) | 1.677 fg | 1.633 ef | 24.803 i | 25.800 e |
|  | (Co1) | 2.110 ef | 2.137 e | 22.933 ij | 22.333 fg |
|  | (Co2) | 2.187 ef | 2.077 e | 22.527 j | 22.300 fg |
|  | (Bs) | 2.413 e | 2.153 e | 22.603 j | 21.333 gh |
|  | (Co1+ Bs) | 2.087 ef | 2.150 e | 21.080 jk | 21.850 g |
|  | (Co2+ Bs) | 2.027 ef | 2.190 e | 20.253 k | 18.850 hi |
| (S2) | (Cont.) | 0.910 hi | 0.983 fg | 41.967 bc | 36.280 c |
|  | (Co1) | 1.100 h | 1.267 f | 34.447 g | 32.970 d |
|  | (Co2) | 1.140 h | 1.283 f | 34.023 g | 33.467 cd |
|  | (Bs) | 1.130 h | 1.347 f | 36.540 f | 33.980 cd |
|  | (Co1+ Bs) | 1.130 h | 1.323 f | 33.657 gh | 31.400 d |
|  | (Co2+ Bs) | 1.190 gh | 1.507 ef | 31.920 h | 24.983 ef |
| (S3) | (Cont.) | 0.563 i | 0.557 g | 51.500 a | 56.600 a |
|  | (Co1) | 0.917 hi | 0.977 fg | 39.693 de | 39.333 b |
|  | (Co2) | 0.943 hi | 1.020 fg | 39.337 de | 40.000 b |
|  | (Bs) | 0.963 hi | 1.210 fg | 43.833 b | 41.933 b |
|  | (Co1+ Bs) | 0.967 hi | 1.187 fg | 40.113 cd | 40.933 b |
|  | (Co2+ Bs) | 1.020 hi | 1.197 fg | 37.783 ef | 39.670 b |
| LSD_0.05_ (SA) | | 0.089 | 0.347 | 0.541 | 0.741 |
| LSD_0.05_ (SP) | | 0.262 | 0.346 | 1.026 | 1.44 |
| LSD_0.05_ (SA × SP) | | 0.525 | 0.693 | 2.052 | 2.88 |

LSD_0.05_ = least significant differences at 0.05 probability. Means with the same letters in the same column are not significantly different (P ≤ 0.05) according to Tukey's test. K:Na ratio and Electrolyte leakage “EL” (%).

**Supplementary Tables S3.**

Influence of applied six salt stress protectants (SP); cobalt (Co1) at 15 ppm, (Co2) at 30 ppm, *Bacillus subtilis* (Bs), (Co1 + Bs), (Co2 + Bs), and distilled water as a control (Cont.) to irrigation water under four levels of NaCl irrigation water salinity (SA); tap water as a control (S0; 0.5, S1; 1.5, S2; 4 and S3; 6 dS m^−1^) and their interaction on Determine the activity of antioxidant enzymes, Ascorbic acid and Proline content in leaf of *Coriandrum sativum* L. cv. Balady. Values are in the form of means of two seasons 2022 and 2023 separate.

| Treatments | | SOD (U/mg protein) | | CAT (U /mg protein) | | MDA (nmol/g f wt^-1^) | | Asco (mg/100 g f wt^-1^) | | Pro (µg /100 g D. wt.) | |
| --- | --- | --- | --- | --- | --- | --- | --- | --- | --- | --- | --- |
|  | | 2022 | 2023 | 2022 | 2023 | 2022 | 2023 | 2022 | 2023 | 2022 | 2023 |
| The main plot (SA) | (S0) | 4.19 D | 4.121 D | 0.316 D | 0.298 D | 0.472 D | 0.508 D | 33.122 A | 32.694 A | 155.962 D | 156.422 D |
|  | (S1) | 4.62 C | 4.522 C | 0.350 C | 0.364 C | 0.984 C | 0.921 C | 29.639 B | 29.078 B | 297.694 C | 289.697 C |
|  | (S2) | 4.93 B | 5.459 B | 0.430 B | 0.464 B | 1.156 B | 1.153 B | 28.356 C | 27.722 C | 534.113 B | 533.407 B |
|  | (S3) | 6.41 A | 6.505 A | 0.563 A | 0.584 A | 1.391 A | 1.413 A | 24.627 D | 26.317 D | 661.858 A | 680.744 A |
| Main effect of subplot (SP) | (Cont.) | 5.22 A | 5.337 A | 0.447 A | 0.456 A | 1.105 A | 1.100 A | 27.775 C | 28.333 C | 450.963 A | 432.591 AB |
|  | (Co1) | 5.05 B | 5.174 B | 0.428 B | 0.438 B | 1.020 B | 1.001 B | 28.508 BC | 28.717 C | 418.064 B | 412.828 BC |
|  | (Co2) | 5.00 BC | 5.108 C | 0.421 BC | 0.427 C | 0.968 D | 0.978 C | 28.875 B | 28.483 C | 422.761 B | 452.153 A |
|  | (Bs) | 5.00 BC | 5.101 C | 0.407 CD | 0.423 CD | 0.992 C | 0.981 C | 28.692 BC | 28.758 BC | 420.405 B | 416.885 B |
|  | (Co1+ Bs) | 4.98 BC | 5.095 CD | 0.401 CD | 0.414 DE | 0.967 D | 0.964 D | 29.242 B | 29.383 B | 384.334 C | 392.378 CD |
|  | (Co2+ Bs) | 4.96 C | 5.063 D | 0.393 D | 0.407 E | 0.943 E | 0.957 D | 30.525 A | 30.042 A | 377.915 C | 383.572 D |
| The interaction between (SA × SP) | | | | | | | | | | | |
| (S0) | (Cont.) | 4.16 l | 4.147 l | 0.290 j | 0.293 l | 0.523 k | 0.517 l | 32.633 bc | 32.033 b | 166.253 j | 163.143 k |
|  | (Co1) | 4.08 l | 4.123 l | 0.320 h-j | 0.297 l | 0.473 l | 0.503 l | 31.900 bc | 32.000 b | 155.019 j | 143.500 k |
|  | (Co2) | 4.18 kl | 4.083 l | 0.320 h-j | 0.300 l | 0.453 l | 0.510 l | 32.267 bc | 32.267 b | 157.935 j | 161.673 k |
|  | (Bs) | 4.22 kl | 4.143 l | 0.340 gh | 0.310 l | 0.467 l | 0.503 l | 32.633 bc | 32.967 ab | 151.297 j | 159.987 k |
|  | (Co1+ Bs) | 4.19 kl | 4.113 l | 0.320 h-j | 0.293 l | 0.467 l | 0.507 l | 33.733 ab | 33.300 ab | 150.904 j | 155.683 k |
|  | (Co2+ Bs) | 4.33 k | 4.113 l | 0.307 ij | 0.293 l | 0.450 l | 0.510 l | 35.567 a | 33.600 a | 154.365 j | 154.547 k |
| (S1) | (Cont.) | 4.82 e-g | 4.690 h | 0.383 ef | 0.407 h | 1.209 d | 1.163 e | 28.967 de | 28.400 d-g | 335.667 g | 306.070 j |
|  | (Co1) | 4.68 g-i | 4.573 i | 0.360 fg | 0.377 i | 1.027 h | 0.915 i | 29.333 de | 29.633 cd | 289.800 hi | 278.010 j |
|  | (Co2) | 4.55 ij | 4.500 j | 0.350 gh | 0.367 ij | 0.903 j | 0.857 k | 29.700 de | 28.367 d-g | 293.400 hi | 286.750 j |
|  | (Bs) | 4.58 h-j | 4.490 j | 0.340 gh | 0.353 jk | 0.963 i | 0.883 j | 29.333 de | 28.400 d-g | 301.800 h | 286.107 j |
|  | (Co1+ Bs) | 4.56 ij | 4.470 jk | 0.343 gh | 0.340 k | 0.920 ij | 0.863 jk | 29.700 de | 29.267 c-e | 278.100 i | 272.483 j |
|  | (Co2+ Bs) | 4.52 j | 4.410 k | 0.325 hi | 0.340 k | 0.883 j | 0.847 k | 30.800 cd | 30.400 c | 287.400 hi | 308.763 j |
| (S2) | (Cont.) | 5.24 c | 5.678 d | 0.513 c | 0.503 e | 1.283 c | 1.285 d | 26.400 fg | 26.933 h-l | 601.933 d | 585.603 ef |
|  | (Co1) | 5.03 d | 5.490 e | 0.453 d | 0.477 f | 1.180 de | 1.158 ef | 28.233 ef | 27.667 f-i | 547.373 e | 550.550 fg |
|  | (Co2) | 4.92 de | 5.428 ef | 0.437 d | 0.460 fg | 1.120 fg | 1.127 gh | 29.333 de | 27.300 g-k | 552.810 e | 593.287 ef |
|  | (Bs) | 4.84 ef | 5.407 fg | 0.393 e | 0.453 g | 1.150 ef | 1.135 fg | 28.600 e | 27.333 g-j | 560.063 e | 518.297 gh |
|  | (Co1+ Bs) | 4.83 e-g | 5.402 fg | 0.393 e | 0.443 g | 1.120 fg | 1.113 gh | 28.233 ef | 28.167 e-h | 475.479 f | 495.230 hi |
|  | (Co2+ Bs) | 4.71 f-h | 5.347 g | 0.390 ef | 0.445 g | 1.080 g | 1.102 h | 29.333 de | 28.933 d-f | 467.021 f | 457.477 i |
| (S3) | (Cont.) | 6.67 a | 6.833 c | 0.600 a | 0.620 a | 1.407 a | 1.437 a | 23.100 i | 25.967 lm | 700.000 a | 675.547 bc |
|  | (Co1) | 6.41 b | 6.510 b | 0.577 ab | 0.600 b | 1.400 ab | 1.427 a | 24.567 g-i | 25.567 m | 680.063 b | 679.250 bc |
|  | (Co2) | 6.36 b | 6.423 c | 0.553 b | 0.583 bc | 1.396 ab | 1.420 ab | 24.200 hi | 26.000 k-m | 686.900 ab | 766.900 a |
|  | (Bs) | 6.32 b | 6.370 c | 0.553 b | 0.573 c | 1.386 ab | 1.400 b | 24.200 hi | 26.333 j-m | 668.460 b | 703.150 b |
|  | (Co1+ Bs) | 6.33 b | 6.396 c | 0.546 b | 0.580 c | 1.363 ab | 1.373 c | 25.300 gh | 26.800 i-m | 632.853 c | 646.116 cd |
|  | (Co2+ Bs) | 6.28 b | 6.383 a | 0.550 b | 0.550 d | 1.357 b | 1.370 c | 26.400 fg | 27.233 g-l | 602.873 d | 613.500 de |
| LSD_0.05_ (SA) | | 0.0469 | 0.035 | 0.009 | 0.014 | 0.019 | 0.0048 | 0.507 | 0.509 | 6.207 | 25.076 |
| LSD_0.05_ (SP) | | 0.0752 | 0.0331 | 0.0165 | 0.009 | 0.022 | 0.013 | 0.932 | 0.654 | 9.223 | 22.512 |
| LSD_0.05_ (SA × SP) | | 0.1505 | 0.066 | 0.0331 | 0.0191 | 0.045 | 0.026 | 1.864 | 1.309 | 18.446 | 45.024 |

LSD_0.05_ = least significant differences at 0.05 probability. Means with the same letters in the same column are not significantly different (P ≤ 0.05) according to Tukey's test. superoxide dismutase activities “SOD” (U/mg protein), Catalase activities “CAT” (U /mg protein), Malondialdehyde “MDA” (nmol g f wt ^-1^), Ascorbic acid “Asco” (mg/100 g f wt ^-1^), and Proline “Pro” (µg /100 g D. wt.).

**Supplementary Tables S4.**

Influence of applied six salt stress protectants (SP); cobalt (Co1) at 15 ppm, (Co2) at 30 ppm, *Bacillus subtilis* (Bs), (Co1 + Bs), (Co2 + Bs), and distilled water as a control (Cont.) to irrigation water under four levels of NaCl irrigation water salinity (SA); tap water as a control (S0; 0.5, S1; 1.5, S2; 4 and S3; 6 dS m^−1^) and their interaction on important biological phytochemicals in seed oil of *Coriandrum sativum* L. cv. Balady. Values are in the form of means of two seasons 2022 and 2023 separate.

| Treatments | | Linalool (%) | | γ-terpinene (%) | | α-pinene (%) | | p-cymene (%) | |
| --- | --- | --- | --- | --- | --- | --- | --- | --- | --- |
|  | | 2022 | 2023 | 2022 | 2023 | 2022 | 2023 | 2022 | 2023 |
| The main plot (SA) | (S0) | 71.96 A | 72.06 A | 3.98 D | 3.98 C | 2.04 D | 2.03 D | 2.85 D | 2.23 C |
|  | (S1) | 59.41 B | 61.45 B | 4.25 C | 4.11 B | 2.62 C | 2.53 C | 3.33 C | 2.69 B |
|  | (S2) | 45.32 C | 52.14 C | 4.51 B | 3.91 D | 3.23 B | 2.80 B | 3.51 B | 2.75 B |
|  | (S3) | 41.91 D | 43.32 D | 4.89 A | 4.73 A | 5.60 A | 5.39 A | 3.77 A | 3.46 A |
| Main effect of subplot (SP) | (Cont.) | 50.84 D | 55.18 D | 4.51 A | 4.12 A | 3.60 A | 3.29 A | 3.45 A | 2.69 C |
|  | (Co1) | 53.56 C | 56.33 C | 4.39 BC | 4.14 A | 3.33 B | 3.13 BC | 3.37 BC | 2.73 C |
|  | (Co2) | 54.37 C | 56.67 C | 4.40 B | 4.21 A | 3.36 B | 3.20 A-C | 3.40 B | 2.76 BC |
|  | (Bs) | 55.52 B | 57.78 B | 4.40 BC | 4.21 A | 3.32 B | 3.17 A-C | 3.34 CD | 2.82 AB |
|  | (Co1+ Bs) | 56.02 B | 58.19 B | 4.39 BC | 4.21 A | 3.41 B | 3.27 AB | 3.31 D | 2.84 AB |
|  | (Co2+ Bs) | 57.59 A | 59.32 A | 4.35 C | 4.20 A | 3.19 C | 3.07 C | 3.30 D | 2.87 A |
| The interaction between (SA × SP) | | | | | | | | | |
| (S0) | (Cont.) | 69.73 b | 72.22 a | 4.05 j | 3.91 e-g | 2.29 hi | 2.18 gh | 2.86 j | 2.22 h |
|  | (Co1) | 72.27 a | 72.26 a | 3.97 jk | 3.97 d-g | 1.93 jk | 1.93 hi | 2.83 j | 2.21 h |
|  | (Co2) | 71.61 ab | 71.97 a | 3.98 jk | 3.96 d-g | 2.03 jk | 2.01 hi | 2.91 j | 2.19 h |
|  | (Bs) | 72.27 a | 72.26 a | 4.01 jk | 4.01 c-f | 2.03 jk | 2.03 hi | 2.81 j | 2.23 h |
|  | (Co1+ Bs) | 72.27 a | 71.83 a | 3.98 jk | 4.00 c-g | 2.12 ij | 2.14 g-i | 2.81 j | 2.26 h |
|  | (Co2+ Bs) | 73.59 a | 71.80 a | 3.90 k | 4.00 c-g | 1.82 k | 1.87 i | 2.86 j | 2.28 h |
| (S1) | (Cont.) | 54.45 e | 58.30 e | 4.33 gh | 4.04 c-e | 2.82 e | 2.64 b-f | 3.50 d-f | 2.61 g |
|  | (Co1) | 57.49 d | 58.48 e | 4.25 hi | 4.17 c | 2.55 fg | 2.50 ef | 3.36 gh | 2.73 d-g |
|  | (Co2) | 59.07 d | 60.35 d | 4.27 hi | 4.18 c | 2.62 e-g | 2.57 d-f | 3.35 gh | 2.77 d-f |
|  | (Bs) | 61.38 c | 62.95 c | 4.26 hi | 4.15 cd | 2.59 fg | 2.53 ef | 3.28 hi | 2.73 d-g |
|  | (Co1+ Bs) | 61.38 c | 63.54 bc | 4.21 i | 4.06 c-e | 2.68 ef | 2.59 c-f | 3.24 i | 2.69 d-g |
|  | (Co2+ Bs) | 62.70 c | 65.05 b | 4.18 i | 4.03 c-e | 2.44 gh | 2.35 fg | 3.22 i | 2.63 fg |
| (S2) | (Cont.) | 40.59 kl | 48.86 j | 4.57 d | 3.80 g | 3.45 c | 2.86 bc | 3.61 c | 2.63 fg |
|  | (Co1) | 42.57 i-k | 50.92 i | 4.56 de | 3.82 fg | 3.20 d | 2.68 b-e | 3.54 c-e | 2.67 e-g |
|  | (Co2) | 45.21 h | 51.56 hi | 4.52 d-f | 3.97 d-g | 3.22 d | 2.82 b-d | 3.57 cd | 2.75 d-g |
|  | (Bs) | 46.53 gh | 52.92 gh | 4.50 d-f | 3.96 d-g | 3.18 d | 2.79 b-e | 3.48 d-f | 2.80 de |
|  | (Co1+ Bs) | 47.52 fg | 53.61 fg | 4.46 ef | 3.95 d-g | 3.25 cd | 2.89 b | 3.46 ef | 2.82 de |
|  | (Co2+ Bs) | 49.50 f | 54.96 f | 4.43 fg | 3.99 c-g | 3.05 d | 2.76 b-e | 3.42 fg | 2.85 d |
| (S3) | (Cont.) | 38.61 l | 41.32 m | 5.07 a | 4.73 ab | 5.84 a | 5.46 a | 3.82 a | 3.30 c |
|  | (Co1) | 41.91 jk | 43.65 l | 4.79 c | 4.60 b | 5.63 ab | 5.41 a | 3.76 ab | 3.31 c |
|  | (Co2) | 41.58 jk | 42.80 lm | 4.85 bc | 4.71 ab | 5.57 b | 5.41 a | 3.76 ab | 3.33 c |
|  | (Bs) | 41.91 jk | 42.99 l | 4.84 bc | 4.72 ab | 5.47 b | 5.33 a | 3.81 ab | 3.53 b |
|  | (Co1+ Bs) | 42.90 ij | 43.78 l | 4.93 b | 4.82 a | 5.57 b | 5.46 a | 3.74 ab | 3.59 ab |
|  | (Co2+ Bs) | 44.55 hi | 45.47 k | 4.89 bc | 4.79 ab | 5.43 b | 5.32 a | 3.71 b | 3.70 a |
| LSD_0.05_ (SA) | | 0.748 | 0.504 | 0.031 | 0.06 | 0.064 | 0.083 | 0.056 | 0.069 |
| LSD_0.05_ (SP) | | 1.154 | 0.792 | 0.054 | 0.101 | 0.112 | 0.145 | 0.049 | 0.077 |
| LSD_0.05_ (SA × SP) | | 2.309 | 1.58 | 0.109 | 0.202 | 0.223 | 0.291 | 0.098 | 0.155 |

LSD_0.05_ = least significant differences at 0.05 probability. Means with the same letters in the same column are not significantly different (P ≤ 0.05) according to Tukey's test. Linalool “Linalool” (%), γ-terpinene “γ-terpinene” (%), α-pinene “α-pinene” (%), and p-cymene “p-cymene” (%)

**Follow - Supplementary Tables S4.**

Influence of applied six salt stress protectants (SP); cobalt (Co1) at 15 ppm, (Co2) at 30 ppm, *Bacillus subtilis* (Bs), (Co1 + Bs), (Co2 + Bs), and distilled water as a control (Cont.) to irrigation water under four levels of NaCl irrigation water salinity (SA); tap water as a control (S0; 0.5, S1; 1.5, S2; 4 and S3; 6 dS m^−1^) and their interaction on important biological phytochemicals in seed oil of *Coriandrum sativum* L. cv. Balady. Values are in the form of means of two seasons 2022 and 2023 separate.

| Treatments | | Camphor (%) | | Geranyl acetate (%) | | Protein (%) | |
| --- | --- | --- | --- | --- | --- | --- | --- |
|  | | 2022 | 2023 | 2022 | 2023 | 2022 | 2023 |
| The main plot (SA) | (S0) | 2.23 D | 2.23 C | 1.82 D | 1.82 C | 22.651 A | 22.188 A |
|  | (S1) | 2.79 C | 2.69 B | 2.18 C | 2.11 B | 21.022 B | 21.221 B |
|  | (S2) | 3.17 B | 2.75 B | 2.44 B | 2.12 B | 19.056 C | 19.296 C |
|  | (S3) | 3.57 A | 3.46 A | 2.75 A | 2.66 A | 16.424 D | 17.595 D |
| Main effect of subplot (SP) | (Cont.) | 2.95 AB | 2.69 C | 2.35 A | 2.15 A | 18.42 C | 19.07 D |
|  | (Co1) | 2.91 BC | 2.73 C | 2.30 B | 2.16 A | 19.87 B | 20.19 BC |
|  | (Co2) | 2.90 C | 2.76 BC | 2.28 B | 2.18 A | 19.95 B | 19.95 C |
|  | (Bs) | 2.96 A | 2.82 AB | 2.31 AB | 2.21 A | 20.06 B | 20.30 B |
|  | (Co1+ Bs) | 2.96 A | 2.84 AB | 2.29 BC | 2.19 A | 20.06 B | 20.29 B |
|  | (Co2+ Bs) | 2.97 A | 2.87 A | 2.25 C | 2.17 A | 20.37 A | 20.65 A |
| The interaction between (SA × SP) | | | | | | | |
| (S0) | (Cont.) | 2.29 h | 2.22 h | 1.85 i | 1.81 e | 22.667 ab | 21.793 b-d |
|  | (Co1) | 2.20 h | 2.21 h | 1.82 i | 1.82 e | 22.730 ab | 22.333 ab |
|  | (Co2) | 2.21 h | 2.19 h | 1.82 i | 1.80 e | 22.483 b | 22.063 a-c |
|  | (Bs) | 2.23 h | 2.23 h | 1.87 i | 1.87 e | 22.960 a | 22.437 a |
|  | (Co1+ Bs) | 2.24 h | 2.26 h | 1.83 i | 1.84 e | 22.540 ab | 22.127 a-c |
|  | (Co2+ Bs) | 2.22 h | 2.28 h | 1.73 j | 1.77 e | 22.523 ab | 22.377 ab |
| (S1) | (Cont.) | 2.79 fg | 2.61 g | 2.27 f | 2.12 b-d | 20.687 e | 20.813 fg |
|  | (Co1) | 2.78 fg | 2.73 d-g | 2.18 g | 2.15 b-d | 20.730 e | 21.210 d-f |
|  | (Co2) | 2.83 f | 2.77 d-f | 2.17 gh | 2.12 b-d | 20.960 de | 21.087 ef |
|  | (Bs) | 2.79 fg | 2.73 d-g | 2.20 fg | 2.14 b-d | 21.293 cd | 21.273 d-f |
|  | (Co1+ Bs) | 2.79 fg | 2.69 d-g | 2.16 gh | 2.09 b-d | 20.980 de | 21.253 d-f |
|  | (Co2+ Bs) | 2.73 g | 2.63 fg | 2.11 h | 2.03 d | 21.480 c | 21.687 c-e |
| (S2) | (Cont.) | 3.17 e | 2.63 fg | 2.46 de | 2.04 cd | 17.437 h | 17.083 k |
|  | (Co1) | 3.20 e | 2.67 e-g | 2.47 d | 2.06 b-d | 19.167 f | 19.313 ij |
|  | (Co2) | 3.14 e | 2.75 d-g | 2.46 de | 2.16 bc | 19.293 f | 18.960 j |
|  | (Bs) | 3.18 e | 2.80 de | 2.46 d | 2.17 b | 19.460 f | 19.917 hi |
|  | (Co1+ Bs) | 3.17 e | 2.82 de | 2.42 de | 2.15 bc | 19.480 f | 20.063 h |
|  | (Co2+ Bs) | 3.16 e | 2.85 d | 2.39 e | 2.15 bc | 19.500 f | 20.437 gh |
| (S3) | (Cont.) | 3.54 c | 3.30 c | 2.83 a | 2.64 a | 12.897 k | 16.587 m |
|  | (Co1) | 3.45 d | 3.31 c | 2.73 bc | 2.63 a | 16.853 ij | 17.897 k |
|  | (Co2) | 3.42 d | 3.33 c | 2.71 c | 2.63 a | 17.043 hi | 17.690 kl |
|  | (Bs) | 3.62 bc | 3.53 b | 2.74 bc | 2.67 a | 16.543 j | 17.583 kl |
|  | (Co1+ Bs) | 3.66 b | 3.59 ab | 2.74 bc | 2.68 a | 17.230 hi | 17.730 k |
|  | (Co2+ Bs) | 3.78 a | 3.70 a | 2.78 ab | 2.72 a | 17.980 g | 18.083 lm |
| LSD_0.05_ (SA) | | 0.062 | 0.069 | 0.037 | 0.049 | 0.244 | 0.480 |
| LSD_0.05_ (SP) | | 0.042 | 0.077 | 0.035 | 0.059 | 0.237 | 0.306 |
| LSD_0.05_ (SA × SP) | | 0.084 | 0.155 | 0.070 | 0.119 | 0.475 | 0.612 |

LSD_0.05_ = least significant differences at 0.05 probability. Means with the same letters in the same column are not significantly different (P ≤ 0.05) according to Tukey's test. Camphor “Camphor” (%), Geranyl acetate “Geranyl acetate” (%), and Protein (%) “protein” (%).

**Supplementary Tables S5.**

Influence of applied six salt stress protectants (SP); cobalt (Co1) at 15 ppm, (Co2) at 30 ppm, *Bacillus subtilis* (Bs), (Co1 + Bs), (Co2 + Bs), and distilled water as a control (Cont.) to irrigation water under four levels of NaCl irrigation water salinity (SA); tap water as a control (S0; 0.5, S1; 1.5, S2; 4 and S3; 6 dS m^−1^) and their interaction on yield parameters of seed and oil of *Coriandrum sativum* L. cv. Balady. Values are in the form of means of two seasons 2022 and 2023 separate.

| Treatments | | SYP (g) | | LO (%) | | EOYS (%) | |
| --- | --- | --- | --- | --- | --- | --- | --- |
|  | | 2022 | 2023 | 2022 | 2023 | 2022 | 2023 |
| The main plot (SA) | (S0) | 3.44 A | 3.42 A | 0.118 C | 0.128 C | 0.60 A | 0.59 A |
|  | (S1) | 3.24 B | 3.23 B | 0.135 B | 0.137 B | 0.52 B | 0.52 B |
|  | (S2) | 2.98 C | 2.61 C | 0.146 A | 0.154 A | 0.48 C | 0.49 C |
|  | (S3) | 1.87 D | 2.01 D | 0.151 A | 0.158 A | 0.45 D | 0.31 D |
| Main effect of subplot (SP) | (Cont.) | 2.74 E | 2.62 F | 0.141 A | 0.153 A | 0.47 C | 0.42 D |
|  | (Co1) | 2.83 D | 2.72 E | 0.138 A | 0.142 BC | 0.50 B | 0.46 C |
|  | (Co2) | 2.84 D | 2.76 D | 0.138 A | 0.148 AB | 0.51 B | 0.48 B |
|  | (Bs) | 2.93 C | 2.86 C | 0.134 A | 0.140 C | 0.51 B | 0.48 B |
|  | (Co1+ Bs) | 2.99 B | 2.95 B | 0.133 A | 0.144 BC | 0.53 A | 0.51 A |
|  | (Co2+ Bs) | 3.05 A | 2.99 A | 0.141 A | 0.141 BC | 0.55 A | 0.52 A |
| The interaction between (SA × SP) | | | | | | | |
| (S0) | (Cont.) | 3.36 bc | 3.33 cd | 0.123 e-h | 0.127 hi | 0.55 cd | 0.54 c-f |
|  | (Co1) | 3.44 ab | 3.39 bc | 0.120 f-h | 0.130 g-i | 0.59 b | 0.57 b-e |
|  | (Co2) | 3.41 b | 3.41 b | 0.117 gh | 0.130 g-i | 0.59 b | 0.59 a-c |
|  | (Bs) | 3.44 b | 3.42 b | 0.120 f-h | 0.127 hi | 0.59 b | 0.58 a-d |
|  | (Co1+ Bs) | 3.45 ab | 3.42 b | 0.120 f-h | 0.130 g-i | 0.63 a | 0.62 ab |
|  | (Co2+ Bs) | 3.53 a | 3.52 a | 0.110 h | 0.123 i | 0.64 a | 0.63 a |
| (S1) | (Cont.) | 3.15 g-i | 3.14 f | 0.137 b-f | 0.147 c-f | 0.49 e-h | 0.49 fg |
|  | (Co1) | 3.20 f-h | 3.23 e | 0.137 b-f | 0.140 e-h | 0.50 ef | 0.51 e-g |
|  | (Co2) | 3.21 e-g | 3.22 e | 0.132 d-g | 0.143 d-g | 0.52 de | 0.52 ef |
|  | (Bs) | 3.27 d-f | 3.23 e | 0.133 c-g | 0.130 g-i | 0.52 de | 0.50 fg |
|  | (Co1+ Bs) | 3.29 c-e | 3.27 de | 0.137 b-f | 0.133 f-i | 0.55 cd | 0.54 c-f |
|  | (Co2+ Bs) | 3.31 cd | 3.32 d | 0.133 c-g | 0.127 hi | 0.56 bc | 0.57 b-e |
| (S2) | (Cont.) | 2.74 m | 2.31 k | 0.143 a-d | 0.163 ab | 0.44 i | 0.40 hi |
|  | (Co1) | 2.92 l | 2.48 j | 0.140 b-e | 0.147 c-f | 0.463 g-i | 0.46 gh |
|  | (Co2) | 2.98 kl | 2.56 i | 0.150 a-c | 0.153 b-e | 0.483 e-h | 0.526 d-f |
|  | (Bs) | 3.06 jk | 2.69 h | 0.150 a-c | 0.153 b-e | 0.47 f-i | 0.53 c-f |
|  | (Co1+ Bs) | 3.08 ij | 2.78 g | 0.153 ab | 0.157 b-d | 0.486 e-h | 0.54 c-f |
|  | (Co2+ Bs) | 3.12 h-j | 2.82 g | 0.140 b-e | 0.153 b-e | 0.50 ef | 0.50 fg |
| (S3) | (Cont.) | 1.72 p | 1.71 n | 0.160 a | 0.173 a | 0.41 j | 0.27 l |
|  | (Co1) | 1.77 p | 1.78 m | 0.153 ab | 0.150 b-e | 0.44 i | 0.29 l |
|  | (Co2) | 1.75 p | 1.84 m | 0.153 ab | 0.163 ab | 0.46 hi | 0.31 kl |
|  | (Bs) | 1.96 o | 2.10 l | 0.133 c-g | 0.150 b-e | 0.45 i | 0.32 j-l |
|  | (Co1+ Bs) | 2.14 n | 2.31 k | 0.153 ab | 0.157 b-d | 0.46 hi | 0.36 i-k |
|  | (Co2+ Bs) | 2.23 n | 2.31 k | 0.150 a-c | 0.160 a-c | 0.486 e-h | 0.37 ij |
| LSD_0.05_ (SA) | | 0.046 | 0.028 | 0.011 | 0.005 | 0.0094 | 0.020 |
| LSD_0.05_ (SP) | | 0.045 | 0.03 | 0.008 | 0.007 | 0.017 | 0.028 |
| LSD_0.05_ (SA × SP) | | 0.09 | 0.07 | 0.0174 | 0.014 | 0.034 | 0.057 |

LSD_0.05_ = least significant differences at 0.05 probability. Means with the same letters in the same column are not significantly different (P ≤ 0.05) according to Tukey's test. Seed yield per plant “SYP” (g), Leaf oil “LO” (%), Essential oil yield “EOYS” (%).
